# Inducible Antibacterial Activity in the Bacillales by Triphenyl Tetrazolium Chloride

**DOI:** 10.1038/s41598-020-62236-z

**Published:** 2020-03-27

**Authors:** Laura Sierra-Zapata, Javier C. Álvarez, Magally Romero-Tabarez, Mark.W. Silby, Matthew F. Traxler, Scott W. Behie, Rita de Cassia Pessotti, Valeska Villegas-Escobar

**Affiliations:** 1 Research group CIBIOP, Department of Biological Sciences, Universidad EAFIT, Medellín, Antioquia, Colombia; 2 Escuela de Biociencias, Universidad Nacional de Colombia, Antioquia, Colombia; 3 Department of Biology, University of Massachusetts Dartmouth, Dartmouth, MA USA; 4 Department of Plant and Microbial Biology, University of California at Berkeley, Berkeley, CA USA

**Keywords:** Metabolomics, Antimicrobials, Applied microbiology, Bacteria, Pathogens, Gene expression

## Abstract

The world is in the midst of an antimicrobial resistance crisis, driving a need to discover novel antibiotic substances. Using chemical cues as inducers to unveil a microorganism’s full metabolic potential is considered a successful strategy. To this end, we investigated an inducible antagonistic behavior in multiple isolates of the order Bacillales, where large inhibition zones were produced against *Ralstonia solanacearum* only when grown in the presence of the indicator triphenyl tetrazolium chloride (TTC). This bioactivity was produced in a TTC-dose dependent manner. *Escherichia coli* and *Staphylococcus* sp. isolates were also inhibited by *Bacillus* sp. strains in TTC presence, to a lesser extent. Knockout mutants and transcriptomic analysis of *B. subtilis* NCIB 3610 cells revealed that genes from the L-histidine biosynthetic pathway, the purine, pyrimidine *de novo* synthesis and salvage and interconversion routes, were significantly upregulated. Chemical space studied through metabolomic analysis, showed increased presence of nitrogenous compounds in extracts from induced bacteria. The metabolites orotic acid and L-phenylalaninamide were tested against *R. solanacearum, E. coli, Staphylococcus* sp. and *B. subtilis*, and exhibited activity against pathogens only in the presence of TTC, suggesting a biotransformation of nitrogenous compounds in *Bacillus* sp. cells as the plausible cause of the inducible antagonistic behavior.

## Introduction

Since the discovery of penicillin^1^ and its subsequent large-scale production and introduction as a therapeutic agent^2^, disease control in both animals and plants has been mainly achieved by the use of natural products (NPs)^3,4^. Antimicrobials constitute a large fraction of these NPs, used to combat animal infections of bacterial origin and to control fungal or bacterial plant diseases^5,6^. Applications other than disease control have resulted in excessive and indiscriminate use of antimicrobials, with reports suggesting that around 80% of antimicrobial use is for non-therapeutic purposes in the US^7,8^ and 70% in the European Union countries^9^. Even though microbial resistance to bioactive NPs occurs naturally^10^, the excessive use of antibiotics selects resistance elements in bacterial populations and makes them available to be captured by previously antibiotic-sensitive pathogens^11^. This is believed to have precipitated the present-day global crisis of antimicrobial resistance (AMR). This crisis has become so severe that the World Health Organization (WHO) and the Food and Agriculture Organization (FAO) from the United Nations (UN) have started synergistic initiatives to counteract the growing appearance of resistant strains by promoting research programs worldwide^12,13^. This underscores the importance of discovery of novel antibiotic substances and antimicrobial technologies for use against pathogens of clinical, veterinary and agricultural importance^14^.

Antibiotics have been historically discovered and sourced from microbes^4,6^, with the soil microbiome being a well-known source of antimicrobial-producing bacteria and fungi. In many bacteria, significant portions of the genome are devoted to the production of NPs, also known as secondary metabolites, with some bacterial isolates having the potential to produce as many as 50 secondary metabolites^6^. These metabolites were traditionally considered to have a natural defense role for the producing organism, being mostly produced under stressful circumstances to selectively inhibit the growth of competitors^15^. However, recent research suggests that there may be alternative or additional roles for antimicrobials. For example, they may act as signaling or quorum sensing molecules and influence regulation of gene expression in natural ecosystems^16,17^. The probable functional diversity of these metabolites indicates that we need a broader understanding regarding the ecological context in which these small molecules are made, how they function in natural settings, and/or how they evolved^16^.

In modern drug discovery and development programs, most compounds currently in the clinical pipeline are modifications of existing classes of antibiotics and represent only short-term solutions to the growing resistance problem. There is a growing interest in the discovery of new families of compounds with novel structures and mechanisms of action that can be used to treat drug resistant pathogens^18^. This interest has been aided by the emergence of a set of innovative technical platforms and methodologies for discovering novel antimicrobials of microbial origin^4,18^. Among these platforms, new cultivation techniques to explore the diverse but yet to be cultured bacteria in soil are a major focus. Cultivation of bacteria is highly biased toward a few phylogenetic groups, many of which exclude currently underexplored bacterial lineages likely to hold novel biosynthetic gene clusters (BGCs) and thus, many biochemical pathways remain to be discovered^19^. The new cultivation approaches have yielded encouraging results, leading to the discovery of two promising new structures, lassomycin^20^ from *Lentzea kentuckyensis* and teixobactin^21^ from *Eleftheria terrae*. An alternative and complementary approach is the investigation of silent BGCs and their activation through chemical cues and microbial interactions^22^. This method has its grounds in the greater metabolic capacity of microorganisms reflected in their genome sequences than that which is expressed in traditional fermentation conditions. A term for this greater capacity is “OSMAC” (one-strain many compounds) and it suggests the activation of unexpressed BGCs through varying growth conditions or cues from other microbes^22,23^. These cues can be of natural origin if they are produced by a neighboring, interacting strain through co-culture, or external and of synthetic origin, achieved by adding individual or mixtures of compounds to the culture media^24^. The recent discovery of Amycomicin^25^ exemplifies the power of chemical enhancement and microbial interactions for unveiling novel metabolites. Further exploration of the total chemical space of those interactions, through metabolomics, can yield a complete spectrum of novel chemistries produced by a given strain during interactions or under inducing conditions^4^.

Here, we describe a system to find bioactive molecules in Bacillales, by causing an alteration in the electron and carbon flow within metabolic pathways using an unusual but well-known chemical cue, triphenyl tetrazolium chloride (TTC), at sub-inhibitory doses. This alteration in culture media triggers an induced antimicrobial activity in the Bacillales. TTC is a mild antibiotic for Gram-positive bacteria and a redox indicator and mediator compound^26^ which readily enter cells, where is reduced by NADPH-dependent oxidoreductases and dehydrogenases into insoluble TPF (triphenyl formazan)^27,28^.

We show that sub-inhibitory doses of TTC induce the production of compounds by Bacilliales which are active against plant pathogens of high economic importance in inter-tropical zones, such as *R. solanacearum* which attacks over 50 plant families^29^, and against mammalian pathogens such as *E. coli* and *Staphylococcus* sp. These compounds are produced by a plausible biotransformation mechanism between TTC and nitrogenous compounds overexpressed in *Bacillus* sp.

## Results

### TTC induces antimicrobial activity in several species of Bacillales against *R. solanacearum* in a dose dependent manner

*R. solanacearum* has been routinely grown on BGTA medium for pathogenomic studies^30^ while King B, LB and TSA media, have often been used to screen antagonist bacteria against *R. solanacearum*^31^. These media all contain complex nutrients but differ in the content of TTC salt. Here BGTA medium was used to evaluate antibacterial activity of 100 isolates from different species of the Bacillales order. Surprisingly, all isolates were inhibitory toward *R. solanacearum*. To determine whether antimicrobial activity was associated with TTC amendment, different isolates (Supplementary Table S1) were tested by the agar plug diffusion test against *R. solanacearum*. In general, all bacterial strains produced inhibition zones when grown in medium supplemented with TTC (BGTA medium) but not without TTC (BGA medium) with the exception of *B. amyloliquefaciens* EA-CB0959 (Table 1). Inhibition zones in the presence of TTC ranged from 7.3 to 26.8 mm, with *B. cereus*, *B. pumilus*, *B. altitudinis, B. licheniformis, B. thuringiensis, B. coagulans* and *A. palidus* (Table 1) showing highest antimicrobial activity. Using other tetrazolium salts (INT: 2-(4-iodophenyl)-3-(4-nitrophenyl)-5-phenyl-2H-tetrazolium chloride; NBT: Nitrotetrazolium blue, XTT: Sodium 2,3,-bis(2-methoxy-4-nitro-5-sulfophenyl)-5-[(phenylamino)-carbonyl]-2H-tetrazolium) did not produced significant inhibitory activity (Supplementary Fig. S1). Because these results might suggest that TTC sensitized *R. solanacearum*, an antibiogram was performed in the presence and absence of TTC. None of the antibiotics (ampicillin, erythromycin, gentamicin, kanamycin B, norfloxacin, penicillin, rifampicin, streptocycline, sulfamethoxazole) generated larger inhibition halos under TTC conditions (Supplementary Fig. S2), indicating that TTC does not sensitize the pathogen but induces the production of antimicrobial compounds in strains from the Bacillales.

**Table 1:** Effect of TTC on the antimicrobial activity of selected AEFB strains against *R. solanacearum* EAP-009.

Species	Strain	Inhibition zone (mm)	
		0 mg/L TTC	50 mg/L TTC
*B. cereus*	EA-CB1047	0.0 ± 0.0*	22.7 ± 1.4 abc
	EA-CB0012	0.0 ± 0.0*	21.2 ± 0.0
*B. pumilus*	EA-CB0009	0.0 ± 0.0*	22.6 ± 0.4 a
	EA-CB0177	0.0 ± 0.0*	26.8 ± 0.6
*B. megaterium*	EA-CB0185	0.0 ± 0.0*	14.0 ± 0.4 e
	EA-CB1057	0.0 ± 0.0*	15.0 ± 0.0 f
*P. pasadenensis*	EA-CB0840	0.0 ± 0.0*	7.3 ± 0.6
*B. subtilis*	EA-CB0015	4.7 ± 0.8*	16.3 ± 0.0
	EA-CB0575	2.8 ± 0.8*	12.8 ± 2.5
	NCIB 3610	3.2 ± 0.8*	15.5 ± 0.4 cdef
	SMY	0.0 ± 0.0*	15.3 ± 0.5
*B. amyloliquefaciens*	EA-CB0959	18.0 ± 1.3	19.7 ± 0.4 b
*B. altitudinis*	EA-CB1450	0.0 ± 0.0*	21.0 ± 3.5 abcd
	EA-CB0686	0.0 ± 0.0*	16.8 ± 0.5
*B. licheniformis*	ATCC14580	0.0 ± 0.0*	22.4 ± 2.9 abcdef
*B. simplex*	ZK5093	0.0 ± 0.0*	17.2 ± 0.2 cdef
*B. thuringiensis*	ZK5165	0.0 ± 0.0*	19.2 ± 2.8 abcdef
*B. coagulans*	ZK5189	0.0 ± 0.0*	22.0 ± 0.0 abcdef
*B. lentus*	ZK5173	0.0 ± 0.0*	16.2 ± 1.2 cdef
*M. marinus*	ZK5187	0.0 ± 0.0*	13.7 ± 0.0 def
*B. firmus*	ZK5172	0.0 ± 0.0*	15.0 ± 2.0 cdef
*A. palidus*	ZK5191	0.0 ± 0.0*	22.2 ± 2.8 abcdef

Intervals represent standard errors of the mean (n = 3).

*denotes a statistically significant difference (P < 0.05) between the conditions 0 mg/L and 50 mg/L for each strain, by Student t-test. Species sharing letters do not differ statistically in their values at 50 mg/L, by Kruskal-Wallis and Post-hoc Dunn-test for multiple comparisons (*P-value* = 1.52 × 10^−2^). Treatments which do not present letters were tested independently from the group and not included in the statistical test.

To test if TTC induced antimicrobial activity in isolates from other taxonomic families such as Burkholderiales, Pseudomonadales, Enterobacteriales, various strains were tested in plug diffusion tests. Inhibition zones were significantly larger in *S. marcescens* UA-1538 and *B. cepacea* UA-1541; reduced in *D. tsuruhatensis* UA-1537, and TTC had no effect on *P. putida* UA-0095 and *H. seropedicae* UA-1542 (Supplementary Table S2). Compared to Gram-negative microorganisms, the inducible activity obtained in strains from the Bacillales was higher and more broadly spread across different species.

**Figure 1:**
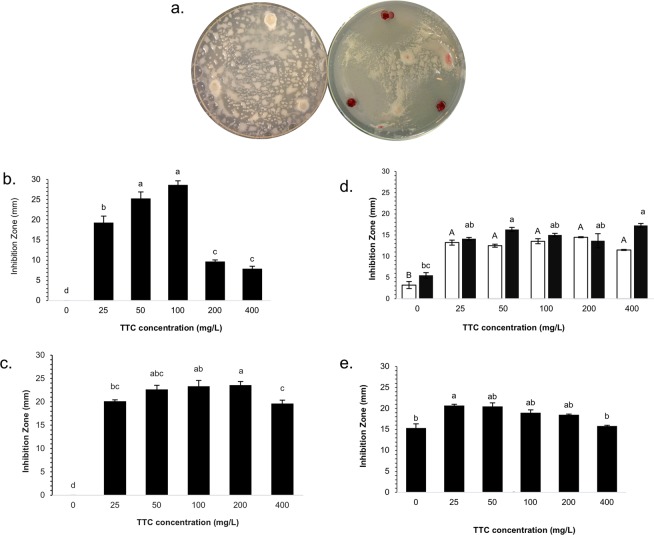
Effect of TTC concentration on inducible antagonism production by *Bacillus* sp. strains. (**a**) Inducible antagonism phenomenon in the presence of TTC of *B. cereus* EA-CB1047 (Left BGA, right BGTA medium). Effect of different TTC concentrations on the size of inducible inhibition zones produced by (**b**) B*. cereus* EA-CB1047, (**c**) *B. pumilus* EA-CB0009, (**d**) *B. subtilis* EA-CB0015 (■) and *B. subtilis* NCIB 3610 (□), and (**e**) *B. amyloliquefaciens* EA-CB0959. Interval bars represent standard errors of the mean (n = 3). Bars sharing capitalized or non-capitalized letters do not differ statistically in their values, determined by ANOVA followed by a Tukey test (for **b**–**d**■ and **e**) and by Kruskal-Wallis methods and post-hoc Dunn test for multiple comparisons (for d□). *B. cereus* EA-CB1047 *P-value* = 4.34 × 10^−11^, *B. pumilus* EA-CB0009 *P-value* = 1.89 × 10^−13^, *B. subtilis* EA-CB0015 *P-value* = 1.19 × 10^−10^, *B. subtilis* NCIB 3610 *P-value* = 8.62 × 10^−2^, *B. amyloliquefaciens* EA-CB0959 *P-value* = 3.30 × 10^−3^).

The size of the inhibition zones was found to be TTC-dose dependent for some strains of Bacillales (Fig. 1b–d) and not dependent on the presence of the target pathogen nor related to nutrient deprivation (Supplementary Fig. S3). Strains *B. cereus* EA-CB1047, *B. pumilus* EA-CB0009, *B. subtilis* NCIB 3610 and *B. subtilis* EA-CB0015, showed an increase in the inhibition zones that were dependent of the TTC dose applied, reaching a maximum between 50 to 100 mg/L (Fig. 1b–d), while *B. amyloliquefaciens* EA-CB0959 inhibition zones were not dose-dependent (Fig. 1e). Above 200 or 400 mg/L of TTC, inhibition zones of *B. cereus* were reduced (Fig. 1b), due to impaired growth at those concentrations (Supplementary Fig. S4a). In contrast, *R. solanacearum* growth was not affected by low-medium (0–100 mg/L) or high (200–400 mg/L) TTC concentrations (Supplementary Fig. S5a). Furthermore, neither *B. cereus*, *B. pumilus* nor *R. solanacearum* growth was impaired in the presence of high concentrations of the TTC salt reduction product TPF (from 0–400 mg/L) (Supplementary Figs. S4b, S4d, S5b). These results altogether suggest that TTC induces the expression of antimicrobial compounds in Aerobic Endospore Forming Bacteria (AEFB) against *R. solanacearum* and possibly against Gram-negative pathogens.

### TTC inducible activity works against other target bacterial species

**Figure 2:**
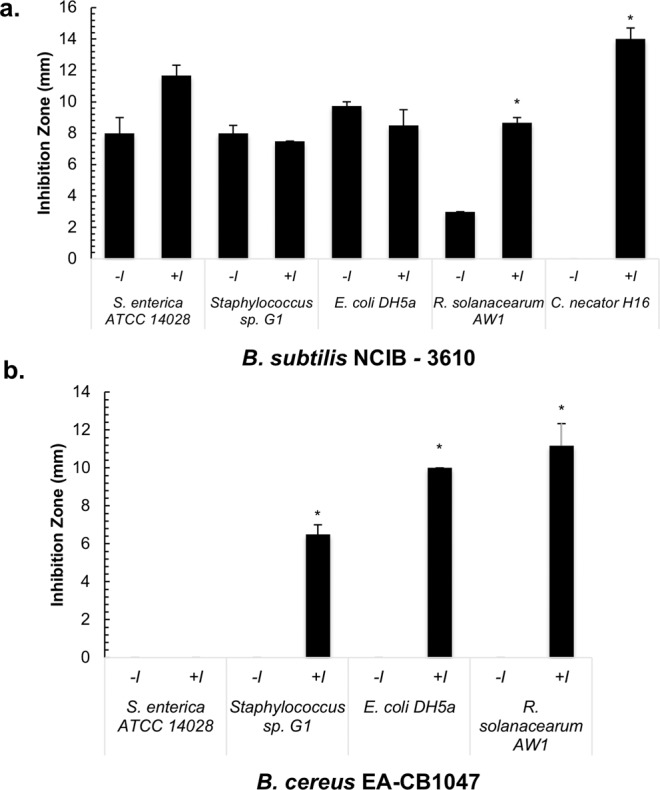
Antagonistic activity of *B. subtilis* NCIB 3610 and *B. cereus* EA-CB1047 against target bacterial strains in the presence and absence of TTC. (**a**) Effect of TTC on the antagonistic activity of *B. subtilis* NCIB 3610 and **(b)** *B. cereus* EA-CB1047 against several target strains. Intervals represent standard errors of the mean (n = 3). +I denotes 50 mg/L TTC amendment in the medium. −I denotes the absence of TTC from the medium. * denotes a statistically significant difference (P < 0.05) between the conditions −I and +I for each antagonistic strain, according to Student t-test ((**a**) *R. solanacearum* AW1 *P-value* = 1.72 × 10^−3^, *C. necator* H16 *P-value* = 2,53 × 10^−3^, (**b**) *Staphylococcus* sp. G1 *P-value* = 2.93 × 10^−3^, *E. coli* DH5α *P-value* = 1.26 × 10^−5^; *R. solanacearum* AW1 *P-value* = 5.37 × 10^−3^).

The spectrum of activity of the inducible antagonism produced by *B. cereus* EA-CB1047 and *B. subtilis* NCIB 3610, was determined against isolates representing diverse genera. Inducible activity was observed against *Cupriavidus necator* for *B. subtilis* NCIB 3610 (Fig. 2a); and *Staphylococcus* sp. and *E. coli* for *B. cereus* EA-CB1047 (Fig. 2b), albeit to a lesser extent than against *R. solanacearum*.

### TTC induces antimicrobial activity of *Bacillus* sp. strains in liquid co-cultures and mono-cultures

To investigate whether TTC-induced antimicrobial activity was restricted to solid cultures, liquid co-cultures were established (Supplementary Fig. S6). We found that in the presence of TTC, the population of *R. solanacearum* decreased 8.3-fold after the onset of stationary phase for *B. cereus*. Similar results were found for *B. subtilis* NCIB 3610 strain (data not shown). Such a pronounced decrease in the population was not observable for *R. solanacearum* in control cultures where TTC was not present. The production of inducible antimicrobial activity in liquid medium, detected through the decrease of *R. solanacearum* colony forming units at approximately 14 h of culture, indicates that inducible compounds can be produced both in liquid and solid media when supplemented with TTC. Although cell-free supernatants of liquid cultures obtained from monocultures of *B. cereus* EA-C1047 grown in the presence of TTC showed activity when sampled between 12–14 h post-inoculation (data not shown), inducible activity was always enhanced on solid media, thus all further tests were done in agar plates, including the extraction of inducible compounds from inhibition zones for metabolomic analysis.

### *B. subtilis* carrying mutations in biosynthetic genes for characterized natural products are not defective in producing inducible inhibition zones

**Figure 3:**
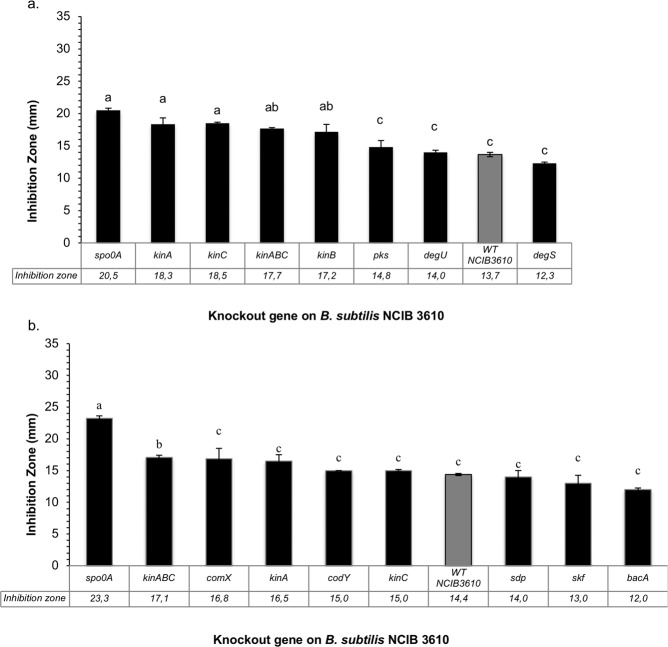
Inhibition zones produced by knockout mutants of *B. subtilis* NCIB 3610 against *R. solanacearum* in BGTA medium. (**a**) *B. subtilis* NCIB 3160 mutants against *R. solanacearum* AW1, (**b**) *B. subtilis* NCIB 3160 mutants against *R. solanacearum* EAP-009. Intervals represent standard errors of the mean (n = 2). Bars sharing non-capitalized letters do not differ statistically in their values, by ANOVA followed by a Tukey test. ((**a**) *P-value* = 1.21 × 10^−4^, (**b**) *P-value* = 2.98 × 10^−3^).

Different *B. subtilis* NCIB 3610 knockout mutant strains defective in the production of well-known antibiotics or in major regulatory pathways (Supplementary Table S3) were tested through the agar plug diffusion test against *R. solanacearum* AW1 and EAP-009 (Fig. 3). No strain was found to be defective, but significantly larger inhibition zones were produced by *B. subtilis* NCIB 3610 mutants *spo0A::erm, kinA::mls, kinB::kan, kinC::cat*. The gene products of *spo0A, kinA, kinB*, *kinC* or downstream expression cascades and metabolic routes regulated by these genes, may be involved in the repression of the inducible antagonism in the WT strain.

### Gene-expression profiling of *B. subtilis* NCIB 3610 cells in presence of TTC shows an upregulation of central genes involved in nitrogen metabolism

**Figure 4:**
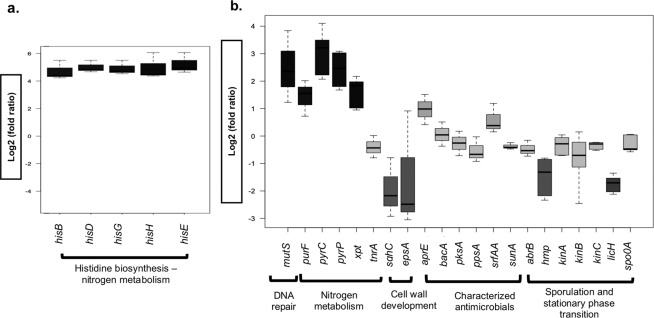
Differential gene-expression profiling of *B. subtilis* NCIB 3610 by two different methodologies under TTC conditions (**a**) total RNA-seq methodology, (**b**) Nanostring. Interval bars represent standard errors of the mean (n = 2).

Transcriptomic profiling obtained by total mRNA-seq and Nanostring both showed expression changes in genes involved in nitrogen metabolism. Specifically, RNA-seq data showed six genes were differentially expressed by TTC-induced *B. subtilis* NCIB 3610 cells relative to controls (log2 of fold-ratio from 4.7 to 5.2), across the three bioinformatic methodologies used (Fig. 4a, Supplementary Tables S4a and S4b). These differentially expressed genes in *B. subtilis* NCIB 3610 were in the major *his* biosynthetic operon (*hisB*, *hisD*, *hisG*, *hisH*, *hisE*) (Fig. 4a). Results of transcriptomic profiling obtained from Nanostring for *B. subtilis* NCIB 3610 (Fig. 4b) showed significant upregulation of genes related to nitrogen metabolism through pyrimidine and purine *de novo* synthesis and salvage and interconversion routes (*pyrP*, *pyrC*, *purF, xpt*), as well as an onset on the DNA damage-machinery (*mutS*), which is related to DNA repair during oxidative stress. On the other hand, downregulated genes were related to exopolysaccharide synthesis (*epsA)* and membrane fluidity and stability (*sqhC*). Consistent with data from mutant studies (above), no induction of genes linked to well-known natural products of *B. subtilis* (bacillaene (*pks*), sublancin (*sunA*), surfactin (*srfAA*), plispastatin (*ppsA*), subtilisin (*aprE*) and bacilysin (*bacA*)) was observed using either technique. Furthermore, when testing the effect of siderophore production by the chromeazurol S (CAS) assay, no increments were found on the size of the inducible inhibition zones (Supplementary Fig. S7).

Overall results for transcriptomic profiling indicate that the two transcriptomic analysis methodologies performed for *B. subtilis* NCIB 3610 cells growing in the presence of TTC converge towards the same induced metabolic route: purine and pyrimidine biosynthesis. All upregulated metabolic pathways are interconnected, with L-histidine and other amino acids such as alanine, glutamate and aspartate being direct precursors for biosynthesis of purine and pyrimidine nitrogenous bases.

### TTC causes alterations in central nitrogen metabolism in *B. subtilis* NCIB 3610

**Figure 5:**
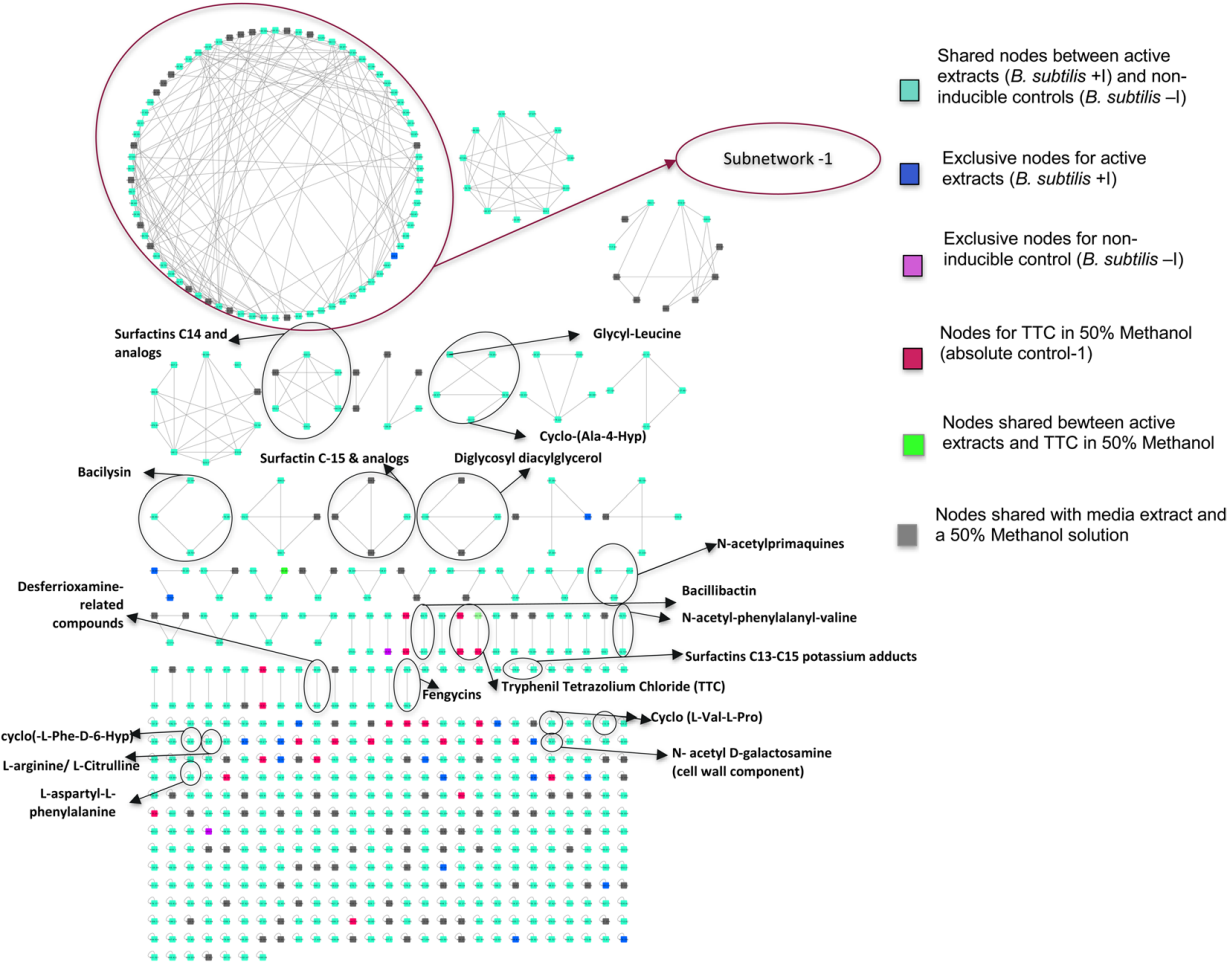
Aggregated and refined spectral network of the metabolomic profile of *B. subtilis* NCIB 3610 extracts. Complete spectral network composed of nodes representing features associated with *B. subtilis* NCIB 3610: active extracts obtained from inducible inhibition zones (blue nodes), extracts from non-induced control (purple nodes), compounds shared between these two extracts (cyan nodes) and other extracts and absolute controls: TTC in 50% methanol (red nodes) and precursor ions shared between control extracts (BGTA medium and 50% methanol solution) and active extracts (grey nodes). These grey nodes denote abundance by more than 2-fold of the precursor ion in active extracts than in control. This figure was created using Cytoscape Version 3.7.1., an open source software platform for visualizing complex networks and integrating these with any type of attribute data. URL: https://cytoscape.org/.

The effect of TTC on the metabolic profile of *B. subtilis* NCIB 3610 is represented in a complex network generated by Cytoscape^32^ using mass spectrometry (MS) data, which were initially analyzed and grouped through the GNPS platform^33^ (Fig. 5). Individual features (precursor ions) present in each treatment are denoted as single nodes within the spectral network, and each node contains one or more MS^2^ spectra which were determined to be identical by the algorithm applied through GNPS platform^33^. The edges connecting nodes denote structural relatedness. The resulting network originally contained 1265 total nodes, distributed in a specific fashion among treatments (Supplementary Table S5).

In the complete network (Fig. 5), several nodes were found to present hits when screened against natural products libraries. These clusters report the presence of surfactins, bacilysin, glycyl-leucine, cyclo-(Ala-4-Hyp), diglycosyl diacylglycerol and N-acetylprimaquines, fengycins, siderophores, bacillibactin, desferrioxamine-related compounds, amino acids, dipeptides and cyclic dipeptides, and N-acetyl-phenylalanyl-valine as well as cell wall components like N-acetyl D-galactosamine and diglycosyl diacylglycerol (Fig. 5, Supplementary Table S6). Interestingly, nitrogenous compounds like amino acids, dipeptides and cyclic dipeptides reported higher spectral counts in the active extract than in the non-induced control extract, as did the N-acetyl primaquines, suggesting an alteration in the nitrogenous intermediates in active extracts. The network includes a pair cluster corresponding to the inducer compound TTC, including a node with the exact inducer compound molecular mass (334.1 + [H]+ = 335.125).

Of particular interest is the subnetwork-1 (Fig. 5) which is made up of 82 nodes representing structurally related unidentified compounds which had 3-fold or higher spectral counts in the active extracts than in the non-induced control. Final selection of nodes for data mining included those in subnetwork-1 which were unique to the active extract or that had at least a 3-fold increase in spectral counts in the active extract. In addition, unclustered features from the larger network classified as unique or only present in the active extract (blue nodes) were included, for a total of 28 selected features (Supplementary Table S7).

Analysis of data mining results suggests that the abundant compounds include selected functional groups associated with intermediates or final products of the nitrogen metabolism biosynthetic routes (Supplementary Table S8). A selection of nitrogen metabolism intermediates and end products which possess the detected functional groups is displayed in Table 2. The list includes compounds such as L-histidine and orotic acid which transcriptomic analysis suggested may be induced. All compounds were validated by comparing the MS^2^ fragmentation pattern of each potential compound to the one of the associated unidentified feature from the network (query).

**Table 2:** Candidate metabolites from metabolomic analysis to which the inducible antagonistic compounds are chemically related.

Names	Chemical classification	CAS Number
L-Arginine Synomym: (s)-2-amino 5- guanidinopentanoic acid	Amino acid	74-79-3
Aicar Synomym: 5-Aminoimidazole-4-carboxamide 1-β-D-ribofuranoside	Adenosine analog	2627-69-2
Quinazoline Synomym: Benzopyrimidine	Aromatic heterocycle	253-82-7
Pyrrole Synomym: Azole, Divinylenimine	Aromatic heterocycle	109-97-7
L-Histidine Synomym: (S)-2-Amino-3-(4-imidazolyl) propionic acid	Amino acid	71-00-1
Asp-Phe Synomym: L-aspartyl-L-phenylalanine, Aspartame acid	Dipeptide	13433-09-5
Gly-Leu Synomym: GLycyl-L-leucine	Dipeptide	869-19-2
Cyclo(L-Phe-trans-4-hydroxy-L-Pro)	Piperazinedione/Diketopiperazines	118477-06-8
Orotic acid Synomym: 2,6-Dioxo-1,2,3,6-tetrahydro-4-pyrimidinecarboxylic acid	Heterocyclic compound	50887-69-9
Imidazole Synomym: 1,3-Diaza-2,4-cyclopentadiene, Glyoxaline	Aromatic heterocycle	288-32-4
Glycinamide Synomym: 2-Aminoacetamide	Amide derivative	598-41-4
L-Phenylalaninamide	Amide derivative	5241-58-7
2-Aminobenzoate	Rare amino acid	118-92-3
Indol Synomym: 2,3-Benzopyrrole	Aromatic heterocycle	120-72-9
Pyrimidine Synomym: 1,3-Diazine	Aromatic heterocycle	289-95-2
1,1-Dimethylguanidine	Amidines and ureas	6145-42-2
Uracil Synomym: 2,4(1 H,3 H)-pyrimidinedione	Pyrimidine derivative	66-22-8

### Compounds of nitrogen metabolism inhibit pathogens sensitive to inducible antagonism, in the presence of TTC

Metabolites from Table 2, specifically L-arginine, L-histidine, imidazole, orotic acid, L-phenylalanylamide, and 1,1-dimethylguanidine were tested at concentrations of 100 µM, 100 mM and 1 M against *R. solanacearum* EAP-009, and strains of *Staphylococcus* sp.*, E. coli*, *B. subtilis* NCIB 3610, *Xanthomonas* sp. and *S. enterica* (Table 3)*. R. solanacearum* growth was inhibited by all six compounds in the presence of TTC at concentrations of 1 M, and by orotic acid at 100 mM. In the absence of TTC, *R. solanacearum* was only inhibited by L-arginine and 1,1-dimethylguanidine at concentration of 1 M. *Staphylococcus* sp. cells were sensitive to five compounds, excluding 1,1-dimethylguanidine, at 1 M in the presence of TTC (Table 3). *E. coli* DH5α was the least sensitive to the nitrogenous compounds, being inhibited only by L-arginine in both the presence and absence of TTC, and by imidazole and L-phenylalaninamide at concentrations of 1 M with TTC present. *B. subtilis* cells were inhibited by all tested compounds with the exception of orotic acid and L-phenylalaninamide. These two compounds became of special interest by being active against the pathogens but not against the producing strain. From results (Table 3), it can be concluded that the inhibitory activity of these compounds either occurs only the presence of TTC or it is significantly higher when the inducer compound is present.

**Table 3:** Candidate compounds from metabolomic analysis show antibiotic activity against target strains in TTC presence.

Compound name	Target species	Inhibition zone (mm)			
		1 M		100 mM	
		50 mg/L TTC	0 mg/L TTC	50 mg/L TTC	0 mg/L TTC
L-Arginine	*R. solanaceraum* EAP-009	16.7 ± 0.45	14.9 ± 0.43	0.0 ± 0.0	0.0 ± 0.0
	*Staphylococcus* sp. G	13.0 ± 0.52*	0.0 ± 0.0	0.0 ± 0.0	0.0 ± 0.0
	*E. coli* DH5α	9.4 ± 0.24	9.0 ± 0.12	0.0 ± 0.0	0.0 ± 0.0
	*B. subtilis* NCIB 3610	13.7 ± 0.84*	0.0 ± 0.0	0.0 ± 0.0	0.0 ± 0.0
L-Histidine	*R. solanaceraum* EAP-009	13.0 ± 0.12*	0.0 ± 0.0	0.0 ± 0.0	0.0 ± 0.0
	*Staphylococcus* sp. G	13.6 ± 0.37*	0.0 ± 0.0	0.0 ± 0.0	0.0 ± 0.0
	*E. coli* DH5α	0.0 ± 0.0	0.0 ± 0.0	0.0 ± 0.0	0.0 ± 0.0
	*B. subtilis* NCIB 3610	10.0 ± 0.48*	0.0 ± 0.0	0.0 ± 0.0	0.0 ± 0.0
Imidazole	*R. solanaceraum* EAP-009	13.3 ± 1.85*	0.0 ± 0.0	0.0 ± 0.0	0.0 ± 0.0
	*Staphylococcus* sp. G	16.7 ± 0.92*	0.0 ± 0.0	0.0 ± 0.0	0.0 ± 0.0
	*E. coli* DH5α	10.0 ± 0.54*	0.0 ± 0.0	0.0 ± 0.0	0.0 ± 0.0
	*B. subtilis* NCIB 3610	20.0 ± 0.44*	0.0 ± 0.0	0.0 ± 0.0	0.0 ± 0.0
Orotic acid	*R. solanaceraum* EAP-009	49.3 ± 1.90*	0.0 ± 0.0	26.5 ± 0.7*	0.0 ± 0.0
	*Staphylococcus* sp. G	12.2 ± 0.15*	0.0 ± 0.0	0.0 ± 0.0	0.0 ± 0.0
	*E. coli* DH5α	0.0 ± 0.0	0.0 ± 0.0	0.0 ± 0.0	0.0 ± 0.0
	*B. subtilis* NCIB 3610	0.0 ± 0.0	0.0 ± 0.0	0.0 ± 0.0	0.0 ± 0.0
L-Phenylalaninamide	*R. solanaceraum* EAP-009	15.0 ± 0.89*	0.0 ± 0.0	0.0 ± 0.0	0.0 ± 0.0
	*Staphylococcus* sp.	14.8 ± 0.34*	0.0 ± 0.0	0.0 ± 0.0	0.0 ± 0.0
	*E. coli* DH5α	10.3 ± 0.31*	0.0 ± 0.0	0.0 ± 0.0	0.0 ± 0.0
	*B. subtilis* NCIB 3610	0.0 ± 0.0	0.0 ± 0.0	0.0 ± 0.0	0.0 ± 0.0
1,1-Dimethylguanidine	*R. solanaceraum* EAP-009	33.7 ± 1.76*	20.7 ± 2.51	0.0 ± 0.0	0.0 ± 0.0
	*Staphylococcus* sp. G	0.0 ± 0.0	0.0 ± 0.0	0.0 ± 0.0	0.0 ± 0.0
	*E. coli* DH5α	0.0 ± 0.0	6.7 ± 0.38	0.0 ± 0.0	0.0 ± 0.0
	*B. subtilis* NCIB 3610	11.5 ± 0.5*	0.0 ± 0.0	0.0 ± 0.0	0.0 ± 0.0

Intervals represent standard errors of the mean (n = 3). Other target strains (*Xanthomonas* sp. and *S. typhimium*) evidenced no sensitivity to any of the tested compounds. No activity was observed when compounds were tested at concentrations below 100 mM.

*denotes a statistically significant difference (P < 0.05) between the conditions 0 mg/L and 50 mg/L of TTC for each strain at a certain concentration of the compound, according to Student t-test.

Although tested compounds showed activity in high concentrations (100 mM−1M) which would not be suitable for *in vivo* applications, these nitrogenous substances alone are probably not the direct product of the inducible phenomenon. Instead, these results suggest that an enzymatic biotransformation of nitrogenous compounds (i.e. orotic acid, L-phenylalaninamide) is occurring with TTC or its reduced product TPF, which might be an incomplete reaction, meaning that reactants are not completely converted to products. Thus, smaller quantities of the nitrogenous products might be needed to observe bioactivity in an *in vivo* system. These products which result from reaction with TTC/TPF are suggested to have bioactivity against *R. solanacearum* and against other target pathogens (e.g. *Staphylococcus* sp., and *E. coli*), and are being studied in detail to elucidate their complete chemical structure.

## Discussion

Isolates from the order Bacillales, which includes the genus *Bacillus*, are well known to produce bioactive substances, with some of these compounds being extensively studied for their *in vitro* and *in vivo* biological activities including anti-fungal, anti-inflammatory and anti-tumor properties^34,35^. But can novel compounds, with diverse and exceptional properties, still be found in widely explored microbial orders such as actinomycetes^36^ and Bacillales^34^?. A promising research avenue is to study the chemistry of microbes within their environments and while interacting with chemical signals^37^, in order to increase the likelihood of finding novel metabolites active against antibiotic resistant pathogens, as well as to inhibit crop pathogens^5^. The research presented here exemplifies this type of alternative platform for discovering active biomolecules. Results here indicate that Bacillales strains generate inducible antagonistic compounds against *R. solanacearum*, *E. coli* and *Staphylococcus* sp. (Figs. 1 and 2) by a process of induction and a possible biotransformation (Table 3). Using wild isolates and type strains (Supplementary Table S1) belonging to several species from the Bacillales order, this research represents innovation in natural product investigations, specifically the capacity of TTC to change the redox balance within the cells and act as an elicitor of bioactivity in the Bacillales.

The production of inducible inhibition zones by *Bacillus* sp. against *R. solanacearum* occurs in a TTC dose-dependent fashion (Fig. 1), a phenomenon previously observed in several antibiosis-inducible species when using chemical signals, specially antibiotics^24^, acting as elicitors or inducers of otherwise silent BGCs in microbial genomes. Various reports support the role of the chemical environment surrounding bacteria as a trigger of metabolic responses, with the term OSMAC illustrating how the metabolic output of a strain can be finely tuned or stimulated in the presence of natural or synthetic signals^22–24^. Some of the most recently reported novel bioactive substances have been isolated either from a chemical interaction with a neighboring microbial strain or natural signal^25^ or by enrichment cultivation methods which concentrate the chemical ecological signals and allow the growth of otherwise non-cultivatable bacteria^21,38^. Tetrazolium salts, particularly the widely used compound TTC, have not been reported as inducers of antimicrobial behavior, and thus this study reveals a novel platform of induction of bioactive substances in microbial strains.

TTC does not induce antibacterial activity by every bacterium. The inducible inhibition zones observed in this study, produced by bacterial isolates in the presence of TTC, are more strongly produced by species from the Bacillales order (Table 1, Fig. 1), than by species from other taxonomic families as Burkholderiales, Pseudomonadales, Enterobacteriales (Supplementary Table S2). These results indicate an intrinsic capacity of *Bacillus* sp. and related species to be more susceptible to metabolite induction than other taxonomical orders tested, with respect to the production of these specific antimicrobial substances in the presence of TTC. Among bacteria there is a high diversity of dehydrogenases and respiratory systems^39^, and it is possible that not all dehydrogenase systems are capable of using TTC to the same extent, becoming toxic to some bacteria at high concentrations (>500 mg/L)^26^ while allowing an enhanced capacity of *Bacillus* sp. to metabolize TTC. This could occur since TTC readily enters the cells, mostly driven by the partial negative charge inside cell membranes due to membrane potential phenomenon^27^, and its intracellular reduction is fundamentally done by membrane bound respiratory chain dehydrogenases. It was then hypothesized that Reactive Oxygen Species (ROS), well known for their cell damaging effects in bacteria^40^, could be overproduced due to the enhanced reductive activity occurring inside *Bacillus* sp. cells and thus be responsible for affecting *R. solanacearum* growth. However, complementation of culture media with antioxidants compounds (α-tocopherol, uric acid, ascorbic acid, L-glutathione) did not alter the size of inhibitions zones produced by *B. cereus* EA-CB 1047 and *B. subtilis* NCIB 3610 against *R. solanacearum* (Supplementary Fig. S8).

Four other traits of the inducible antagonism observed in the presence of TTC support the conclusion that a chemical interaction with TTC is the triggering mechanism for producing the observed antibiosis: first, the induction is TTC dose-dependent (Fig. 1), with the largest inducible inhibition zones observed within the range of 50–100 mg/L for all *Bacillus* species tested. Second, TTC does not sensitize the pathogen *R. solanacearum* to any from a wide range of antibiotic classes (Supplementary Fig. S2). Third, the antagonism test using polyamide membranes shows that nutrient depletion is not the cause of observed inhibition zones and excludes a possible microbial interaction with the target pathogen (Supplementary Fig. S3). Finally, when knockout mutant stains from B. *subtilis* NCIB 3610 were tested for inducible antibiosis capacity (Fig. 3) none was repressed in the production of TTC-induced inhibition zones. Given that the majority of these knockout mutants were in genes related to biosynthesis of known natural products such as surfactins and bacilysin, it can be suggested that these known compounds are not directly responsible for the observed growth impairment of target strains and are apparently not being induced, and thus TTC likely induces production of other inhibitory compounds.

When the system was investigated using gene expression analysis, a significant upregulation of the nitrogen metabolism (Fig. 4, Supplementary Tables S4a and S4b) was observed from two different methodologies employed: total RNA-seq and Nanostring. This observation does not correlate with other transcriptomic findings for antimicrobial-producing Bacillales strains: for example, Yang *et al*.^41^ studied antibiosis activity of *B. subtilis* induced by a neighboring *B. cereus* strain and found that most overexpressed genes were related to glycolysis and the TCA cycle. Another study looked at a plant-growth-promoting (PGP) strain of *B. subtilis* (OKB105) from a transcriptomics perspective during interaction with its host plant, where surfactin production is a key factor in its protective effects. That study revealed several overexpressed metabolic routes, specifically carbohydrate metabolism, processes of transport/binding and lipoproteins and RNA regulation^42^.

Among some of the most interesting results of the present research is the convergence of results between the two transcriptomic approaches, RNA-seq and Nanostring, which both indicate that the L-histidine, purine and pyrimidine biosynthetic routes were highly upregulated in *B. subtilis* NCIB 3610 cells in inducible conditions (Fig. 4, Supplementary Table S4). L-histidine and other amino acids such as alanine, glutamate and aspartate are direct precursors for purine and pyrimidine nitrogenous base biosynthesis. This can support the transcriptomic results which suggest a common deviation of much of the cell’s pool of excess of nitrogen to these biosynthetic routes, possibly resulting from TPF accumulation. Another possible scenario for this diversion of the cell nitrogen metabolism is an upregulation of amino acid and nitrogenous base biosynthesis, specifically through the *his* biosynthetic operon and purine and pyrimidine salvage and interconversion routes (*pyrP*, *pyrC*, *purF, xpt*), due to an excess of reducing equivalents NAD+ and NADP+ in their oxidized form, produced due to high reduction rates of TTC into TPF.

Besides these findings, gene-expression analysis (Fig. 4) supports results from experiments with knockout mutants and validates the hypothesis that previously characterized natural products are not directly responsible for the inducible antagonism. The expression of known NP biosynthetic genes was not significantly altered by the presence of TTC. There is strong evidence to support the conclusion that characterized NPs produced by *Bacillus* are not the compounds responsible for the inducible inhibition zones produced by *Bacillus* sp. cells in the presence of TTC.

Metabolomic data in subnetwork-1 are supportive of the interpretation of transcriptomic data. The metabolomic data converge on detection of unique or more numerous spectral counts of nitrogenous compounds in extracts from inducible inhibition zones (Fig. 5). The detailed analysis from these nodes, coupled with transcriptomic analysis information, led to the selection of compounds such as L-arginine, L-histidine, imidazole, orotic acid, phenylalaninamide and 1,1-Dimethylguanidine for antagonism tests (Table 2, Table 3). Results from the antagonism tests of these compounds show that most of the metabolites are only active in the presence of TTC and are acting as antimicrobials when in high concentrations. Above values of 100 mM, most of them, which include precursors, intermediates and end-products of biosynthetic routes from nitrogen metabolism, are active against both plant and mammalian pathogens (*R. solanacearum, E. coli* and *Staphyloccous* sp.) (Table 3). Of special interest are orotic acid and L-phenylalaninamide, which both showed activity against the sensitive pathogens but not against the producing strain *B. subtilis*. Results (Table 3) highlight the role of orotic acid, which has previous reports of acting as an antibacterial agent^43^, and has a central role as a precursor of pyrimidine nucleotides^44^.

From overall results we conclude that the overproduction of nitrogen metabolism intermediates is related to the process of intracellular TTC reduction and TPF accumulation. Foundations are set to further explore an enzymatic biotransformation of these nitrogenous intermediates with TTC or TPF, resulting in biologically relevant compounds which are acting as the inducible antimicrobials. Further work, suggesting a plausible mechanism of inhibition, is a priority for this research. Work is currently ongoing in this area by analyzing the genomes of the target pathogen *R. solanacearum* in the search for genes which could code for targets of the compounds, and by determination of the identity and structure of the inhibitory molecule or group of molecules within this pool of bio-transformed nitrogenous compounds.

## Materials and Methods

### Microorganisms

Aerobic Endospore Forming Bacteria (AEFB) isolated from *Musa* sp. plants (Humboldt Institute Collection No. 191), *B. subtilis* NCIB 3610 (wild type, WT), *B. subtilis* SMY, *B. subtilis* PY79 and knockout strains of NCIB 3610 (Supplementary Tables S1 and S3) were used in the different inducible antagonism trials. These strains were stored in TSB (trypticase soy broth, Merck, Germany) with 20% v/v glycerol at −80 °C and activated in TSA (trypticase soy agar, Merck) or LB agar (Luria Bertani Agar, Basingstoke, Oxoid, England) for 48 h at 30 °C before use. Strains *R. solanacearum* EAP-009 (GenBank accession N° KU603426), *Serratia marcescens* EAD-005 (GenBank accession N° KU603427)^45^ and *R. solanacearum* AW1^46^, all were stored in BG medium^47^ plus 20% v/v glycerol at −80 °C and activated at 30 °C for 72 h in BGA medium (BG plus 18 g/L agar (BD, Ontario, Canada)). BG medium was composed of 10 g/L special peptone (Oxoid), 1 g/L casamino acids (BD), 1 g/L yeast extract (Merck) and 5 g/L glucose (Merck). *Pseudomonas putida* UA-0095, *Xanthomonas* sp. UA-1539, *B. cepacea* UA-1541, *S. marcescens* UA-1538, *Salmonella* sp. ATCC 14028, *Staphylococcus* sp. G and *E*. *coli* DH5α (Supplementary Table S1) were stored in TSB (trypticase soy broth, Merck) with 20% v/v glycerol at −80 °C and activated in TSA or LB agar for 48 h at 30 °C before use.

### Inducible antagonism tests

BGTA medium (BGA supplemented with 50 mg/L of TTC (Merck, Darmstadt, Germany)), was used for inducible antagonism tests. BGA medium, without TTC^48^ served as a control. Two methods of antagonism tests^49^ were employed: (i) agar plug diffusion test, to evaluate the inducible antagonism capacity of different bacterial strains against target strains, and (ii) Well-diffusion test, to determine the inducible antagonism capacity of strains belonging to *B. subtilis* NCIB 3610 knockout mutants against two strains of *R. solanacearum*, EAP-009 and AW1 and the antibiotic activity of extracts. Briefly, for the agar plug diffusion test, 100 μL of the target strain at 1 × 10^6^ CFU/mL were plated onto BGTA and BGA plates. In another version, the target strain was submerged into molten BGTA or BGA at a desired final concentration and mixed well. A 0.5 cm plug (diameter) containing the antagonistic strain grown in the appropriate medium (50% TSA, LB) was placed on top of the target strain. After 48 h of incubation at room temperature (22 ± 1 °C), the radius size of inhibition zones were recorded. For the well diffusion test, wells were opened with a sterile Pasteur pipette in BGTA and BGA plates containing the target strain, either submerged or plated, followed by the addition of 50 μL of an overnight inoculum of the antagonistic strain grown at 22 °C and 150 rpm. After incubation at 22 °C for 48 h, the radius of inhibition zones was registered in two equidistant points from the center of the plug or well and an average value was obtained. In each plate, three plugs or wells were made for each treatment and three replicates (plates) per treatment were used. Each experiment was independently repeated two or three times. Variations in the amendments to the culture media, such as tetrazolium salts, TTC concentration, and antioxidants, were done accordingly to evaluate different conditions as outlined in the relevant places in the text.

### Transcriptome analysis

The effect of TTC on the transcriptome profile of *Bacillus* sp. cells was performed by total RNA sequencing (RNA-seq) and Nanostring platform for *B. subtilis* NCIB 3610. For RNA-seq, RNA was extracted from submerged cultures of *B. subtilis* NCIB 3610 grown either in BGT or BG for 14 h at 22 °C and 150 rpm. At 14 h of culture, 500 μL were withdrawn and treated with RNAprotect cell reagent (Qiagen, Netherlands) and then every step of the RNeasy RNA isolation kit (Qiagen, Netherlands) was followed. Total RNA libraries were prepared with kit for Illumina NEBNext (New England Biolabs, MA, USA) and sequenced at TUCF Genomics (Tufts University Core Facility, Boston, MA, USA). Data analysis was carried using an automatic pipeline in Slurm Workload Manager 18.08.1. Differential expression analysis was performed through three approaches: Tuxedo suite, specifically the CuffDiff tool^50^, R-package DE-seq^51^ and EDGE-pro^52^ in order to calculate differential gene expression (DE) through the normalized parameter RPKM (read per million reads per million bases sequenced). Fold change was calculated by the ratio of the RPKM parameter in the presence of TTC vs absence of TTC, and its significance by the statistic p-adj value. Statistical analysis and data visualization prior to data analysis was done using PCA (principal component analysis) and Venn diagrams. PCA was done to asses replicates dispersion and Venn diagrams to find common DE genes across methodologies. Reported values consist of log 2 scale of fold ratios.

For Nanostring, total RNA was extracted from well diffusion assays in BGTA plates and control BGA, from three biological replicates per treatment using RNeasy RNA isolation kit (Qiagen, Netherlands). The experiment was repeated two times independently. Counts for each gene of *B. subtilis* NCIB 3610 were subjected to a technical normalization considering the counts obtained for positive control probe sets. After the technical normalization, a biological normalization was done using housekeeping genes *def*, *folB*, *glpF*, *gsaB*, *ptsG* included in the CodeSet used, *via* the Nanostring nCounter analysis system (Nanostring technologies, USA)^53^. We applied student’s t-test to evaluate the significance of gene expression differences between the TTC supplementation and control using companion software nSolver package (Nanostring technologies, USA). Relative over or under-expression of each gene across different groups was calculated in log2 scale of fold ratio and the level of statistical significance was defined as p ≤ 0.05^54^. Each experiment was independently repeated two times for both methodologies (Nanostring and RNA-seq).

### Metabolomic analysis

The effect of TTC on the metabolic profile of induced *B. subtilis* NCIB 3610 cells was determined by UHPLC/MS. Active and controls extracts were obtained by methanolic extraction of 20 g of agar from inhibition zones by a methodology based on the studies by Liu *et al*.^55^ and Watrous *et al*.^56^ were agar extractions were performed. Briefly, 20 g of agar cut from control and antagonism plates were mixed with 80 mL of methanol in sterile glass flasks, sonicated with 30% amplitude for 20 min and agitated by pulse-vortexing every 20 min for 4 h. The solution was then filtered by 0.2 μm polyamide filter and the solvent evaporated 10X. Control extracts were obtained by extracting 20 g from *B. subtilis* NCIB 3610 grown on BGA plates with no presence of TTC and no *R. solanacearum* inoculation (non-induced control). Negative controls included: media control, obtained by extracting 20 g of BGTA with methanol, inducer control (solution of 50 mg/L of TTC in methanol) and methanol control (50% of aqueous methanol solution). Activity evaluation of each extract followed prior to metabolomic analysis, through agar well diffusion method, using *R. solanacearum* AW1 as target strain.

### Metabolomic data acquisition

Two replicates of each treatment or control were subjected to UHPLC/MS analysis. The equipment for acquiring metabolomic data consisted of an UHPLC system (Dionex UltiMate 3000, ThermoFisher, USA) coupled to a mass spectrometer (MS, Thermo Scientific Q-Exactive Quadrupole-Orbitrap mass spectrometer with a Heated Electrospray Ionization (HESI) source). Sample was injected (5 µL) into a C18 column (50 mm × 2.1 mm, 2.2 µm, Thermo Scientific AcclaimTM RSLC) at 0.4 mL/min and 30 °C. Separation was carried by a gradient of solvent A (water + 0.1% formic acid) and solvent B (methanol + 0.1% formic acid), starting at 10% B for 0–1.5 min, then increasing from 10–100% from 1.5–11.5 min, then at 100% B from 11.5–14.5 min, lowering from 100–10% from 14.5–15 and ending at 10% B from 15–18 min. Peak detection was done at 214 nm on a universal diode array detector, followed by injection into the coupled MS equipment. MS analyses were done in a positive ion mode, with a scan range of 150–2000 *m/z*. Full scan parameters were: resolution of 70,000 full width at half-maximum (FWHM), automatic gain control (AGC) target of 3 × 10^6^ ions and a maximum ion injected time (IT) of 100 ms. The injection capillary temperature was 300 °C, sweep gas was N_2_ and spray voltage was 3.5 kV. MS/MS parameters were: resolution of 17.500 FWHM, AGC target of 1 × 10^5^ ions, maximum IT of 50 ms, quadrupole isolation window of 4.0 *m/z*, normalized collision energy (NCE) of 30%. Tandem MS was acquired using the data-dependent Top5 method considering precursor ion abundance. Data obtained from UHPLC and MS was visualized using Xcalibur software.

### Bioinformatic analysis and spectral networking

Data files (.mzXML) were uploaded to the Mass Spectrometry Interactive Virtual Environment (MassIVE) data repository for further analysis through Global Natural Products Social Molecular Networking (GNPS) web platform^33^. Conditions for GNPS analysis are detailed in Supplementary Table S5. Spectral network (Fig. 5) was generated using data from GNPS and cytoscape. Further network curation and annotation was done manually, through literature reports and AntiSmash analysis^57^ (Supplementary Table S6); the majority of control nodes were removed, leaving only the ones in which the spectral counts from the active extracts outnumbered 3 fold or more the detected spectra from control extracts. *m/z* values of sub-network-1 (Fig. 5) nodes were extracted manually and used as queries on molecular weight search on Chemspider^58^ and PubChem^59^ databases using a source code (Supplementary Table S8).

### Candidate compounds testing

Different compounds derived from metabolomic analysis were tested against target strains, *R. solanacearum*, *Staphylococcus* sp., *E. coli, Xanthomonas* sp., *S. enterica* (Table S1). Specifically, 1 M stocks of: imidazole (Sigma-Aldrich, OH, USA), uric acid (Sigma-Aldrich, OH, USA), L-histidine (Sigma-Aldrich, OH, USA), L-arginine (Sigma-Aldrich, OH, USA), Uracil (Sigma-Aldrich, OH, USA), 1,1-dimethylguanidine (Sigma-Aldrich, OH, USA) orotic acid (Sigma-Aldrich, OH, USA), L-phenylalanylvaline (Sigma-Aldrich, OH, USA) were prepared in appropriate solvents (sterile distilled water, 50% aqueous methanol for 1,1-dimethylguanidine) and filtered through a polyamide 0.2 µm pore-size syringe filter (Sartorius, Gottingen, Germany). Well-diffusion antagonism tests were prepared with each compound reaching final concentrations of 100 µM, 100 mM and 1 M.

### Statistical analysis

All datasets for each experiment were analyzed either in R^60^ or by a Student t-test (TTEST function) when having two condition datasets. As a first step for R package analysis, datasets were tested for their fitting to ANOVA (analysis of variance) assumptions: independence of observations, normal distribution of residuals and homoscedasticity or homogeneity in variance of residuals. If assumptions were met, a one-way ANOVA analysis followed, with an **α** = 0.05 (95% family-wise confidence level), calling statistically significant differences across means of treatments if *P*-value ≤ 0.05. Post-hoc analysis consisted of a Tukey honestly significance difference test in order to find specific differences among groups. If ANOVA assumptions were not met, a Kruskal-Wallis non-parametric test was performed, and homogeneous groups were found if *P-*value ≤ 0.05, by applying Dunn post-hoc method.

## Supplementary information

Supplementary Information.

## Data Availability

The datasets generated during and/or analyzed during the current study are available from the corresponding author on reasonable request.
