# Supplementary material for: Inducible Antibacterial Activity in the Bacillales by Triphenyl Tetrazolium Chloride
Source: Sci Rep. 2020 Mar 27;10:5563. doi: 10.1038/s41598-020-62236-z (PMC7101371; doi:10.1038/s41598-020-62236-z)
Supplement: Supplementary file 1 — Supplementary Information. [file 41598_2020_62236_MOESM1_ESM.pdf]

Inducible Antibacterial Activity in the Bacillales by Triphenyl Tetrazolium Chloride

Supplementary Information Appendix

Laura Sierra-Zapata[1], Javier C. Álvarez[1], Magally Romero-Tabarez[2], Mark. W Silby[3], Matthew F. Traxler[4], Scott W. Behie[4], Rita de Cassia Pessotti[4], Valeska Villegas-Escobar[1*]

[1] Research group CIBIOP, Department of Biological Sciences, Universidad EAFIT, Medellín, Antioquia, Colombia.
[2] Escuela de Biociencias, Universidad Nacional de Colombia, Calle 59A No 63 – 20, Medellín, Antioquia, Colombia.
[3] Department of Biology, University of Massachusetts Dartmouth, Dartmouth, MA, USA.
[4] Department of Plant and Microbial Biology, University of California at Berkeley, Berkeley, CA, USA.
*Corresponding author: vvilleg2@eafit.edu.co

Table S1. Producer and target bacterial strains used in the study.

Species/genus	Strains	Origin	Collection /donator
**Gram positive stains**			
B. cereus	EA-CB 1047	P	Humboldt Institute Collection Nº 191
	EA-CB 0012	R	Humboldt Institute Collection Nº 191
B. pumilus	EA-CB 0009	R	Humboldt Institute Collection Nº 191
	EA-CB 0177	P	Humboldt Institute Collection Nº 191
B. megaterium	EA-CB 0185	R	Humboldt Institute Collection Nº 191
	EA-CB 1057	P	Humboldt Institute Collection Nº 191
Paenibacillus pasadenensis	EA-CB 840	P	Humboldt Institute Collection Nº 191
	EA-CB 0015	P	Humboldt Institute Collection Nº 191
B. subtilis	EA-CB 0575	R	Humboldt Institute Collection Nº 191
	NCIB 3610	N	BGSC
	SMY	N	BGSC
B. amyloliquefaciens	EA-CB 0959	P	Humboldt Institute Collection Nº 191
B. altitudinis	EA-CB1450	P	Humboldt Institute Collection Nº 191
	EA-CB 0686	R	Humboldt Institute Collection Nº 191
B. licheniformis	ATCC14580	So	Kolter Lab strains collection
B. simplex	ZK5093	N	Kolter Lab strains collection
B. thuringiensis **subsp.** ***Darmstadiensis***	ZK5165	N	Kolter Lab strains collection
B. coagulans	ZK5189	S	Kolter Lab strains collection
B. lentus	ZK5173	Si	Kolter Lab strains collection
Marinibacilllus marinus	ZK5187	N	Kolter Lab strains collection
B. firmus	ZK5172	So	Kolter Lab strains collection
Aeribacillus palidus	ZK5191	N	Kolter Lab strains collection
**Gram negative strains**			
Delftia tsuruhatensis	UA-1537	B	Dr. Camilo Ramírez. Universidad de Antioquia
Herbaspirillum seropedicae	UA-1542	B	Dr. Camilo Ramirez. Universidad de Antioquia
Burkholderia cepacea	UA-1541	B	Dr. Camilo Ramirez. Universidad de Antioquia
Pseudomonas putida	UA-0095	B	Dr. Camilo Ramirez. Universidad de Antioquia
Serratia marcescens	UA-1538	B	Dr. Camilo Ramírez. Universidad de Antioquia
**Target strains**			
Xanthomonas **sp.**	UA-1539	B	Dr. Camilo Ramírez. Universidad de Antioquia
Ralstonia solanacearum	AW1	T	Dr. Tim Denny. University of Georgia
Ralstonia solanacearum	EAP09	B	Universidad EAFIT
Escherichia coli	DH5α	CGSC	Universidad EAFIT
Cupriavidus necator **(before** ***R. eutropha)***	H16	DSMZ 428	Dr. Mark Silby, UMass Dartmouth
Staphylococcus **sp.**	G	W	Universidad EAFIT
Salmonella enterica	ATCC 14028	A	Universidad EAFIT
Pectobacterium **sp.**	N	N	Dr. Roberto Kolter, Harvard University

P: phylloplane of banana plants from commercial plantations. R: Rhizosphere of banana plants from commercial plantations. B: Moko infected tissues from banana plants. BGSC: Bacillus Genetic Stock Center, Columbus, OH. DSMZ: Libniz-Institute DSMZ German collection of Microorganisms and Cell cultures. N: Unknown. S: sediments. Si: Symbiont of gram negative *Xenorhabdus*. So: soil.

Table S2. Inducible antagonism capacities of Proteobacteria strains against *R. solanacerum* EAP-009.

Species	Strain	0 mg/L TTC	50 mg/L TTC
		Inhibition zone (mm)	Inhibition zone (mm)
D. tsuruhatensis	UA-1537	13.8 ± 2.1 *	9.5 ± 1.3 b
P. putida	UA-0095	11.6 ± 1.9	11.4 ± 2.8 b
S. marcescens	UA-1538	7.0 ± 0.3	11.8 ± 0.1 b
B. cepacea	UA-1541	0.0 ± 0.0 *	9.3 ± 1.0 b
H. seropedicae	UA-1542	0.0 ± 0.0	0.0 ± 0.0 c
B. cereus	EA-CB1047	0.0 ± 0.0 *	20.8 ± 2.8 a

Intervals represent standard errors of the mean (n=3). * denote a statistically significant difference ($P < 0.05$) between the conditions 0 mg/L and 50 mg/L for each strain, according to Student t-test. Species sharing letters do not differ statistically in their values at 50 mg/L, by one-way ANOVA and Tukey multiple range-test (P-value = 8.61 E-5).

Table S3. *B. subtilis* NCIB-3610 knockout mutant strains

Genotype	Strain code	*knockout gene main function
spo0A::erm	ZK385	Stage 0 sporulation protein
kinA::mls, kinB::kan, KinC::cat	ZK3706	Sporulation kinases A, B, C
KinA::mls	ZK5157	Sporulation kinase A
KinB::cm	ZK5158	Sporulation kinase B
KinC::cm	ZK5159	Sporulation kinase C
degS::tet	ZK3737	Signal transduction histidine-protein kinase/phosphatase DegS
∆***degU***	HV1130	Transcriptional regulatory protein DegU
abrB::tet	ZK4279	Transition state regulatory protein
∆***pksX: specR***	ZK3640	Polyketide synthase involved in bacillaene production
sinI::spc	ZK4200	Transcriptional regulator. Nutrient depletion and development
ppsBΩTn10: specR	ZK3639	Biosynthesis of the lipopeptide antibiotic plipastatin
∆***bacA***	NL363	Synthesis of dipeptide antibiotic bacilysin
srfAA::erm	ZK3858	Surfactin synthase subunit-1
comA::cat srfA-lacZ	ZK3863	Transcriptional regulator (quorum-sensing and competence)
sunA::erm	NL384	Bacteriocin sublancin
eps :: tet tasA :: km comX :: spc	ZK3771	Competence pheromone (quorum-sensing system)
skf :: cm	ZK3748	Sporulation killing factor (toxin)
sdp :: spc	ZK3749	Sporulation delaying protein (toxin)
codY::spc	NL3864	Transcriptional repressor. Nutritional limitation sensor
∆***sigH***	NL161	RNA polymerase sigma-H factor. Transcriptional regulator
∆***sigD***	NL165	RNA polymerase sigma-D factor. Transcriptional regulator
∆***sigE***	NL166	RNA polymerase sigma-E factor Transcriptional regulator
∆***sigX***	NL167	RNA polymerase sigma-X factor Transcriptional regulator
∆***sigY***	NL361	RNA polymerase sigma-Y factor Transcriptional regulator
∆**sigW**	NL362	RNA polymerase sigma-W factor. Transcriptional regulator

*Source: (www.uniprot.org/uniprot/P08874) (1)

Table S4a. Transcriptomic analysis of *B. subtilis* NCIB-3610 in the presence and absence of TTC by RNA-seq technology

**Strain**	**Gene**	**Log 2 (Fold ratio (+I/-I))**					
		**Tuxedo**	**Edge-pro**	**De-seq**	**Average**	**Standard error**	**Biological function***
	BS3610_RS12370	-4,1	-4,3	-4,4	**-4,3**	0,1	Hypothetical protein
	hisH	6,1	4,4	4,5	**5,0**	0,6	Imidazole glycerol phosphate synthase subunit HisH
	hisD	5,5	4,7	4,9	**5,0**	0,3	Histidinol dehydrogenase HisD
B. subtilis	*hisG*	4,7	4,5	5,5	**4,9**	0,3	ATP phosphoribosyltransferase HisG
**NCIB-3610**	*hisH*	ND	4,6	ND	**4,7**	0,0	Imidazole glycerol phosphate synthase subunit HisH
	hisI	6,1	4,6	5,0	**5,2**	0,4	Histidine biosynthesis bifunctional protein HisIE
	hisB	5,5	4,2	4,4	**4,7**	0,4	Imidazoleglycerol-phosphate dehydratase HisB
	BS3610_RS12405	-4,0	-3,9	-4,1	**-4,0**	0,1	Hypothetical protein

Table S4b. Transcriptomic analysis of *B. subtilis* NCIB-3610 in the presence and absence of TTC by for Nanostring technology.

Metabolic pathway	Gene name	Biological function	log2 (fold ratio)
	mutS	DNA damage machinery	2,8
**Nitrogen**	*purF*	purine biosynthesis	1,5
	pyrC	Dihydroorotase from de novo pyrimidine metabolism.	3,0
	pyrP	Uracil transporter from pyrimidine salvage pathway	2,4
	tnrA	Transcriptional repressor, active under N-limitation	-0,4
	xpt	Xanthine phosphoribosyltransferase from purine salvage pathway	1,6
**Development**	*epsA*	Polysaccharide component of matrix	-2,2
	sqhC	Hopanoid metabolism involved in membrane synthesis	-1,9
**Antimicrobials**	*pksA*	Bacillaene production	-0,3
**production**	*sunA*	Sublancin lantibiotic precursor peptide	-0,4
	bacA	Bacilysin; first gene in operon	0,1
	ppsA	Plipastatin synthetase	-0,6
	aprE	Subtilisin E serine alkaline protease	1,0
	srfAA	Surfactin production and competence	0,6
**Sporulation & stationary**	*abrB*	Regulation of transition state genes, pleiotropic	-0,5
**phase general regulators**	*spo0A*	Stage 0 sporulation protein (central role in sporulation)	-0,3
	kinA	Sporulation Kinase A	-0,3
	kinB	Sporulation Kinase B	-0,9
	kinC	Sporulation Kinase C	-0,3
**Other processes**	*hmp*	flavohemoglobin: involved in nitric oxide stress	-1,5
	licH	6-phospho-beta-glucosidase	-1,7

Table S5. GNPS Spectral networking methods and summary

Methodology: data deom .mzXML files was filtered by removing all MS/MS peaks within +/- 17 Da of the precursor m/z. MS/MS spectra were window filtered by choosing only the top 6 peaks in the +/- 50 Da window throughout the spectrum. The data was then clustered with MS-Cluster with a parent mass tolerance of 2.0 Da and a MS/MS fragment ion tolerance of 0.5 Da to create consensus spectra. Further, consensus spectra that contained less than 5 spectra were discarded. A network was then created where edges were filtered to have a cosine score above 0.75 and more than 6 matched peaks. Further edges between two nodes were kept in the network if and only if each of the nodes appeared in each other's respective top 10 most similar nodes. The spectra in the network were then searched against GNPS' spectral libraries. The library spectra were filtered in the same manner as the input data. All matches kept between network spectra and library spectra were required to have a score above 0.7 and at least 6 matched peaks. Analog search was enabled against the library with a maximum mass shift of 100.0 Da. A spectral network was visualized using Cytoscape 3.5 (2), with precursor ion mass as the node value, and m/z difference (deltaMZ parameter) and cosine as the edge values, out of MS spectra obtained from two time-independent experiments. Cytoscape Network was built from GNPS data, and further curation, annotation and group discrimination was enabled by assigning a color for each treatment or group, later clearing nodes from negative control groups. Self-loops, corresponding to non-clustering precursor ions were also displayed. Finally, the generated network was manually curated by filtering nodes which only included files from the two replicates for each treatment. Manual annotation followed, identifying BGC for *B. subtilis* NCIB-3610 by AntiSmash and then identifying clusters in the network which displayed precursor ions with the same MS value (m/z value) as the predicted compound (BGC). Once identified, their MS/MS were analyzed and a match with a literature reported compound was called when one of the precursor ions from the cluster shared the same molecular weight (m/z) $\pm$ 0.5 Da and six or more peaks of its MS/MS spectra with the literature reported compound.

**Parameter**	**Value**
Number of nodes	1.265
Number of pairs	975
Number of ID'd clusternodes	97
Number of ID'd clusternodes not in components	24
Number of connected components identified	31
Number of clusternodes in identified components	288
Number of spectra in consideration	41.062
Number of spectra in network	26.765
Number of ID'd spectra	5.969
Number of ID'd spectra not in components	374
Number of unidentified neighbor spectra	3.021
Number of spectra in identified components	14.498

Nodes: a specific precursor ion with a m/z value associated for which one or more spectra have been identified by MS
Clusternodes: group of nodes connected by edges based on a calculated value (cosine) according to their structural similarity (MS/MS spectra similarity)
Pairs: pairs of nodes
Components: spectral families of compounds (nodes and clusters interconnected), based on their MS/MS spectra similarity

Results from GNPS spectral networking analysis of metabolomic data (https://gnps.ucsd.edu/ProteoSAFe/status.jsp?task=3a62050b552d4281ab438b649c70bd3e) summarize UPLC-MS information obtained from two time-independently obtained active extracts and controls. Given that molecular networks are visual displays of the chemical space present in MS experiments, the statistics of a specific analysis evidence the complexity, purity and other variables such as interrelatedness of compounds and chemical diversity, from the dataset obtained.

Table S6. Manual annotation for metabolic network. Data from GNPS library hits, from *in silico* BGC prediction by Antismash and from reference MS/MS available from literature

Literature				Network		
**Most similar known cluster**	**[M+H]+**	**Reference**	**Ions**	**[M+H]+**	**Ions**	**Shared Peaks**
**Surfactin BGC** (82% of genes show similarity)	1031.75	(3)	Identified by GNPS	1030.64	Identified by GNPS	30
	1059.72			1058.82		26
	1075, 83			1076.64		25
	1081.3	(4)		1081.03		24
	1036.34	Sigma-Aldrich		1036.69		28
**Citrulline BGC** (27% of genes show similarity)	176.2	PubChem	61.1<br>70.1<br>72.7<br>116.2<br>130.2<br>147.2<br>159.1<br>176.2	175.339	60.06<br>70.07<br>72.08<br>116.07<br>130.09<br>147.01<br>159.1<br>176.06	8
**Fengycin BGC** (100% of genes show similarity)	1462.70	(5)	1064.57<br>950.49<br>458.26<br>330.16<br>250.15<br>226.12<br>198.12<br>115.08	1462.54	1063.51<br>949.45<br>457.24<br>330.17<br>249.16<br>226.12<br>198.12<br>115.09	8
	1477.60		1344.78<br>1064.5<br>950.49<br>561.3<br>475.22<br>412.30	1478.59	1343.16<br>1063.5<br>949.44<br>561.27<br>474.27<br>412.22	11

			226.12<br>198.12<br>169.13<br>115.08<br>102.05		226.12<br>198.12<br>169.12<br>115.09<br>102.06	
**Bacillibactin biosynthetic gene cluster**<br>(92% of genes show similarity)	883.2	(6)	883.42<br>587.0<br>313.19	883.79	883.42<br>588.30<br>313.1	7
	883	(7)	295.1<br>293.1<br>249.1<br>191.6	883.79	295.14<br>292.13<br>250.12<br>192.16	
**Teichuronic_acid_biosynthetic_gene_cluster**<br>(100% of genes show similarity)			Identified by GNPS	276.07	Identified by GNPS	6
**N-acetylgalactosamine**	221,089	Chemspider				
**Glucuronic acid**	194,139	Chemspider				
**Bacilysin_BGC**<br>(100% of genes show similarity)	270,285	Pubchem	271.19<br>225.17<br>200.11<br>182.16	270.96	271.1<br>225.1<br>200<br>182.1	6
	270	(8)	165.1<br>136.08		165.1<br>136	

Table S7. Selected nodes for data mining from spectral networking analysis of *B. subtilis* NCIB-3610 extracts

Precursor mass (m/z value) or $[M+H]^+$	Number of spectra for active extract*	Number of spectra for non-induced extract*	Overexpression ratio (qualitative abundance in active extract)
195.092	5	0	5.0
248.149	5	0	5.0
253.019	5	0	5.0
275.151	6	0	6.0
312.107	5	0	5.0
320.161	7	2	3.5
337.152	17	0	17.0
379.664	5	0	5.0
382.635	6	2	3.0
385.403	6	0	6.0
394.947	5	0	5.0
416.403	10	3	3.3
496.455	12	4	3.0
498.141	18	6	3.0
529.298	5	1	5.0
571.897	29	8	3.6
573.369	5	0	5.0
581.510	5	0	5.0
614.881	7	0	7.0
615.525	6	0	6.0
670.408	10	2	5.0
676.192	7	0	7.0
705.338	9	3	3.0
802.000	9	0	9.0
889.417	9	2	4.5
956.418	8	0	8.0
979.159	5	0	5.0
995.998	5	0	5.0

All selected nodes were detected in two time-independently obtained active extracts
$[M+H]^+$ means that molecular mass value of each compound is $[M+H]^+$-1.
Number of spectra for each metabolite were obtained for each condition from GNPS network analysis

Table S8. Subnetwork-1 and unique precursor ions data mining in Chemspider and Pubchem

Methodology: the source code for data mining in chemical databases using precursor mass as entry was the one below. From results, Considerations for calling a match were meeting the following criteria: for mass values, being inside the tolerance interval of maximum ± 5 ppm (maximum 0.01 Da, since ppm means different things at different *m/z* or Da values) of the original compound molecular weight (*m/z*) and for the MS^2 fragmentation pattern, to have at least six matching peaks with the corresponding feature in the spectral network (9).

```
# -*- coding: utf-8 -*-
"""chemspidersearch.ipynb

Automatically generated by Colaboratory.

Original file is located at
    https://colab.research.google.com/drive/1IYZ8tCWb-DmOVysYjXW0mlCHypsqv5eY

#Script to search precursor ions in Chemspider and Pubchem
#by Javier C Alvarez, UEAFIT, 2018
"""

pip install chemspipy

#Libraries
from chemspipy import ChemSpider
from chemspipy import objects
from IPython.display import display, Image

#Token ID
cs = ChemSpider('c306881c-e280-46f8-b91b-559df2b75182')

#Opening data file
masses = ['669.408', '319.161', '528.298', '558.199']
#with open(precursor_maseses.txt) as mass_file:
#  for line in mass_file:
#    masses.append(float(line))

#Definition for searching
def search_by_mass(mass, mass_range, datasources=None, order=None, direction=None):
    qid = cs.filter_mass(mass, mass_range, datasources, order, direction)
    while True:
        status = cs.filter_status(qid)
        if status['status'] == 'Complete':
            csids = cs.filter_results(qid)
            compounds = [objects.Compound(cs, csid) for csid in csids]
            return compounds
        elif status['status'] in {'Failed', 'Unknown', 'Suspended', 'Not Found'}:
            raise Exception('Error searching by mass')

#Searching masses and compounds
for mass, numcomp in zip(masses, range(len(masses))):
  tol = 0.01
  mass_results= cs.search_by_mass(mass, tol)
  print("Searching compund "+ str(numcomp + 1) + "with" + str(len(masses)))
  print("Searching mass of "+ str(mass) + "with tolerance of " + str(tol))

  for compound, counter in zip(mass_results[0:5], range(len(mass_results[0:5]))):
    print("Result " + str(counter + 1))
    imagefilename = str(compound.csid) + ".png"
    with open(imagefilename, "wb") as outimage:
      outimage.write(compound.image)
    print("Compound: " + str(compound.csid))
    print("Molecular formula: " + compound.molecular_formula)
```

```
    print("Molecular weight: " + str(compound.molecular_weight))
    print("SMILES: " + compound.smiles)
    print("Common name: " + compound.common_name)
    print(compound, image_url)
    display(Image(imagefilename))
    print("\n")
  print("\n\n\n")
```

**Molecular Mass $[M+H]^{-1}$ (query)**	**Molecular Mass Hit Compound**	**Selected chemical family / functional groups**
669.408	**669.400** [a]	Aminophenoxy - dibromophenyl
	**669.399** [b]	Quinazoline - Iminomethyl
319.161	**319,161** [a]	Acetylamino - Methylbutanoate
	**319.161** [b]	Tetrazole
528.298	**528.298** [a]	Pyrrol - Amionbenzoate
	**528.298** [b]	Phenylmetilidene-Diazine - Trione
888.417	**888.414** [a]	Imidazol Carboxamide
	**888.416** [b]	Hidrazinylidene
570.897	**570.897** [a]	Glycinamide
	**570.897** [b]	Aminopropanoate
613.881	**613.881** [a]	Carbonohydrazide
	**613.881** [b]	Hexanamide
994.998	**994.998** [a]	Aminohexanoic acid
	**994.998** [b]	Aminophenyl
252.019	**252.020** [a]	Fluoroquinolona
	**252.019** [b]	Pyrimidinylacetamide
311.107	**311.106** [a]	Quinoline
	**311.107** [b]	Fluoroquinoline
274.151	**271.151** [a]	Sulfonamide
	**274.151** [b]	Triazole
336.152	**336,152** [a]	Thiazol
	**336.152** [b]	Indole
378.664	**378.664** [b]	Benzamide
384.403	**384.403** [a]	Aminopentanoate
	**384.403** [b]	Acetamide - Pyrimidine
675.192	**675.193** [a]	L-phenylalaninamide
	**675.192** [b]	Trione
393.947	**393.947** [b]	Methanethione
194.092	**194.093** [a]	Thiocyanate
	**194.092** [b]	Carmamide
955.418	**955.41** [b]	Pyrrol
978.159	**978.159** [b]	Octapeptide
247.149	**274.149** [b]	Dimethylguanidine
580.510	**580.51** [b]	Pyrimidione
572.369	**572,369** [b]	Oxopyrimidine

Letter denotes mined database, a: chempsider, b: pubchem.

SI Figures

Figure S1. Effect of tetrazolium salts INT, NBT and XTT on antagonistic capacity of *B. cereus* EA-CB1047 against *R. solanacearum* EAP-009.

Methodology: The effect of other tetrazolium salts on the inducible activity was tested using the agar plug method. Each Tetrazolium salt was added in a concentration of 50 mg/L to BGA medium. INT (Sigma-Aldrich, OH, USA): 2-(4-iodophenyl)-3-(4-nitrophenyl)-5-phenyl-2H-tetrazolium chloride; NBT (Sigma-Aldrich, OH, USA): Nitrotetrazolium blue, XTT (Sigma-Aldrich, OH, USA): sodium 2,3,-bis(2-methoxy-4-nitro-5-sulfophenyl)-5-[(phenylamino)-carbonyl]-2H-tetrazolium, and TTC (Sigma-Aldrich, OH, USA): 2,3,5-triphenyl-2H-tetrazolium chloride, and were used at different final concentrations (25, 50 and 100 mg/L) on BG agar. Controls for these trials consisted of BGTA and BGA plates without other tetrazolium salts. Three replicates were used for each treatment.

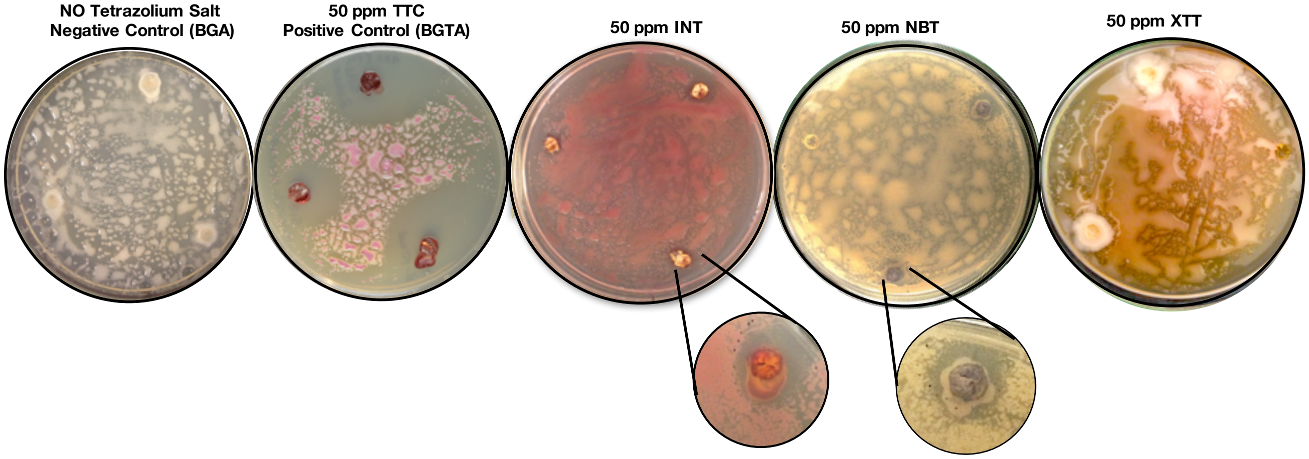

Figure S2. Antibiogram of *R. solanacearum* in presence (BGTA) and absence (BGA) of TTC.

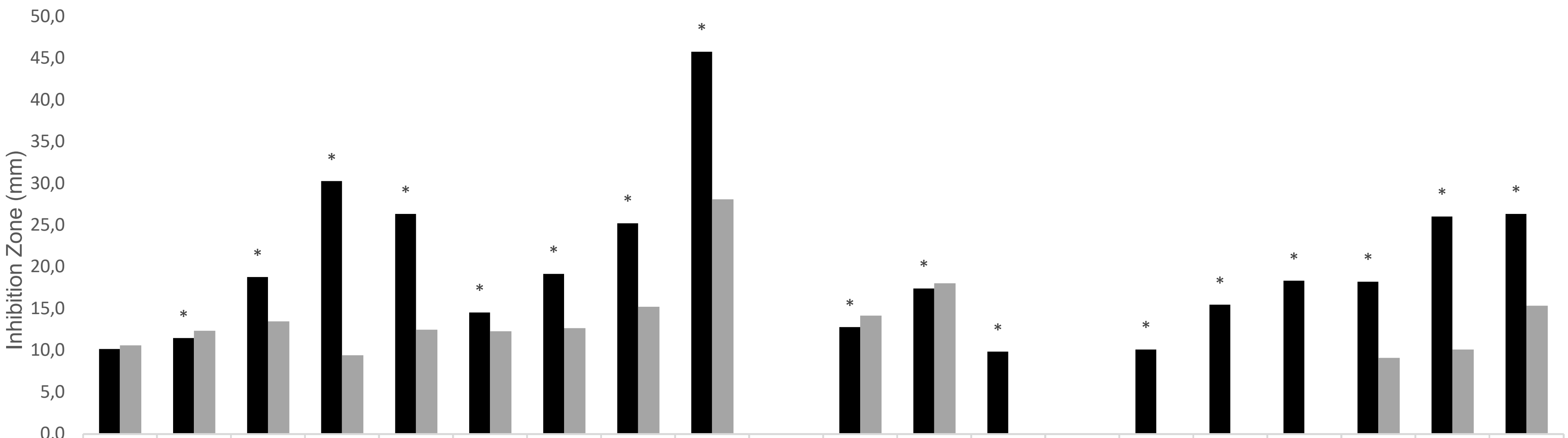

	Ampicillin			Erythromycin	Gentamicin	Kanamycin B			Norfloxacin	Oxytetracycline Hydrochloride			Penicillin	Rifampicin			Streptocycline			Sulphomethoxazole
	C1	C2	C3	C1	C1	C1	C2	C3	C1	C1	C2	C3	C1	C1	C2	C3	C1	C2	C3	C1
BGA	10,2	11,5	18,8	30,3	26,4	14,6	19,2	25,3	45,9	0,0	12,8	17,5	9,9	0,0	10,2	15,5	18,4	18,3	26,1	26,4
BGTA	10,7	12,4	13,5	9,5	12,5	12,3	12,7	15,3	28,2	0,0	14,2	18,1	0,0	0,0	0,0	0,0	0,0	9,2	10,1	15,4

C1, C2 and C3 correspond to concentrations of 10 mg/L, 100 mg/L and 1000 mg/L respectively for each antibiotic. Interval bars represent standard errors of the mean (n = 3).

* denotes statistically significant difference ($p < 0.05$) in the antibiotic effect against target pathogen in BGTA medium (TTC presence) and BGA medium (TTC absence) by Student t-test (TTEST)

Figure S3. Inhibition zones produced by *B. cereus* EA-CB1047 across permeable polyamide membranes against *R. solanacearum*.

Methodology: To determine the ability of *Bacillus* sp. strains to produce the inducible antagonistic activity without the presence of the target pathogen, sterile polyamide (nylon) membrane filter discs (47 mm, 0.2 μm, Sartorius, Gottingen, Germany), were used to set up the agar plug diffusion test on BGTA and BGA plates. Plugs of *Bacillus* sp. previously grown on 50% TSA (Merck, Darmstadt, Germany) for 24 h at 30ºC, where settled on top of the nylon membranes, placed beforehand on the surface of BGA and BGTA plates, and incubated at RT for 48 h. After incubation, membranes where carefully removed and 100 μL of *R. solanacearum* EAP-009 target strain were applied. Plates were additionally incubated for 48 h at RT after which inhibition zones were measured. Positive controls consisted of 1) co-culture of *B. cereus* EA-CB1047 with *R. solanacearum* AW1 by the agar plug diffusion test, 2) *B. cereus* EA-CB1047 grown over the membrane filter disc on a previously *R. solanacearum* inoculated plate. Negative controls included i) *B. cereus* EA-CB1047 grown over the membrane filter disc on BGA plates (without TTC) before plating *R. solanacearum* AW1, ii) a non-inducible strain *H. seropedicae* UA-1542, grown over membrane filter disc in BGTA medium before plating *R. solanacearum* AW1. Three replicates per treatment were used.

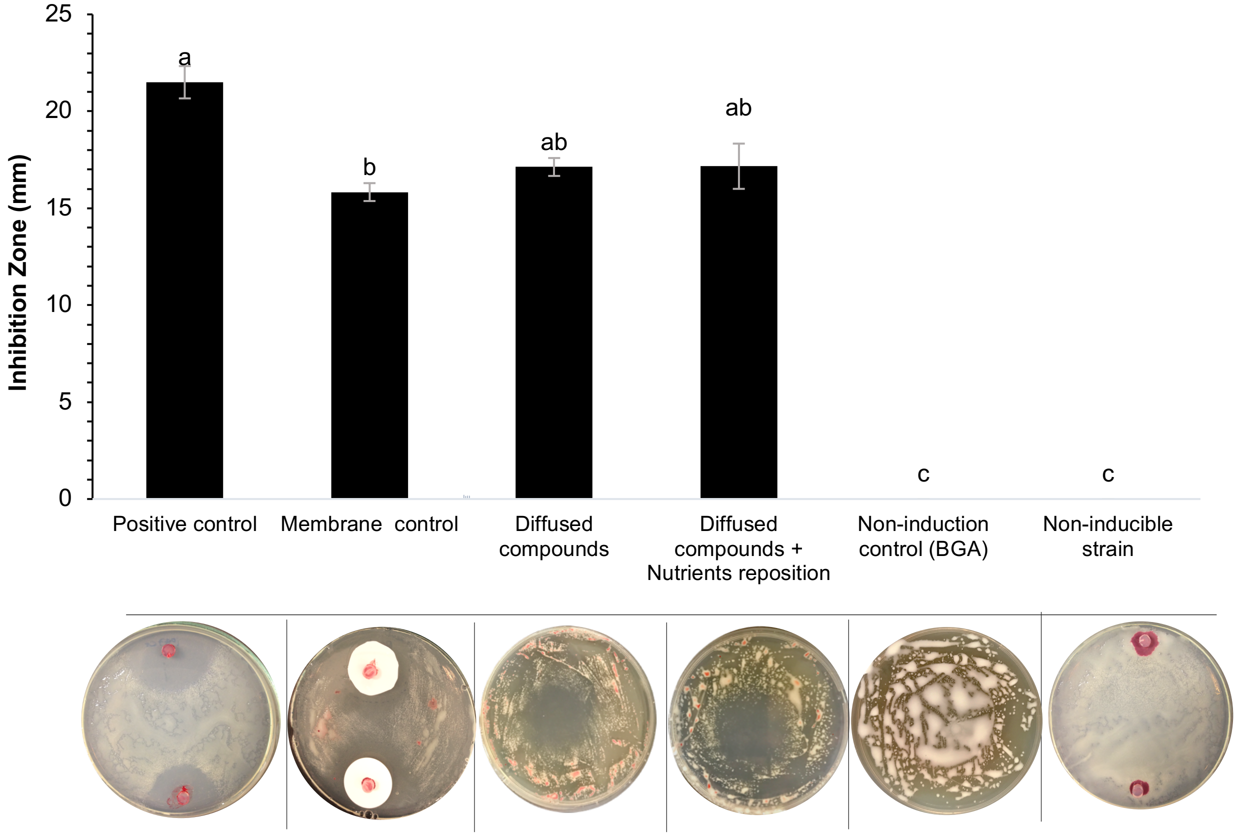

Error bars represent standard errors of the mean (n = 3). Bars sharing letters do not differ statistically in their values, by Kruskal-Wallis rank test and Post-hoc Duncan-test for multiple comparisons (*P-value* = 9.53 E-5). Inhibition zones are indicated with small arrows were occurring. Positive control: co-culture of *B. cereus* EA-CB1047 with *R. solanacearum* AW1 by the agar plug diffusion test. Membrane control (second positive control): *B. cereus* EA-CB1047 grown over the membrane filter disc on a previously *R. solanacearum* inoculated plate. Diffused compounds: *B. cereus* EA-CB1047 growing for 48h over nylon membranes before plating target strain. Diffused compounds + nutrients reposition diffused compounds treatment + 100 μL of concentrated fresh BG medium added were membrane was placed. Non-induction control (BGA, negative control): *B. cereus* EA-CB1047 growing over nylon membranes in a medium without TTC before plating target strain. Non-inducible strain (second negative control): *H. seropedicae* UA-1542 has no antagonistic activity against target strains in BGTA medium.

Figure S4. Effect of different concentrations of TTC and TPF on growth of *B. cereus* EA-CB1047 and *B. pumilus* EA-CB0009. a) TTC – *B. cereus* EA-CB1047, b) TPF - *B. cereus* EA-CB1047, c) TTC – *B. pumilus* EA-CB0009, d) TPF - *B. pumilus* EA-CB0009.

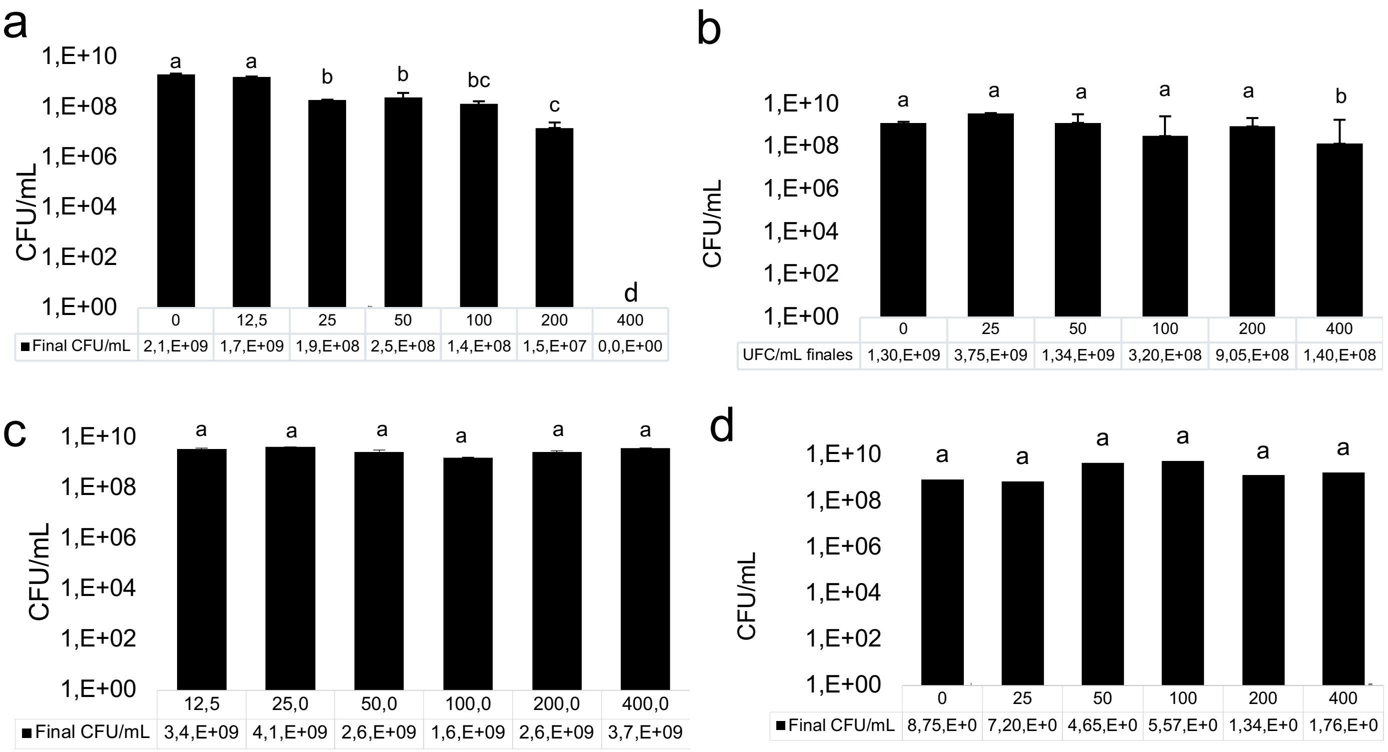

Error bars represent standard errors of the mean (n = 3). Bars sharing letters do not differ statistically in their values detected by Kruskal-Wallis rank test and Post-hoc Duncan-test for multiple comparisons (a) *P- value* = 4.98 E-2 (b) *P- value* = 1.20 E-1 c) *P- value* = 1.38 E-1 d) *P- value* =7.40 E-1)

Figure S5. Effect of different concentrations of TTC and TPF on growth of *R. solanacearum* EAP09. a). TTC, b) TPF.

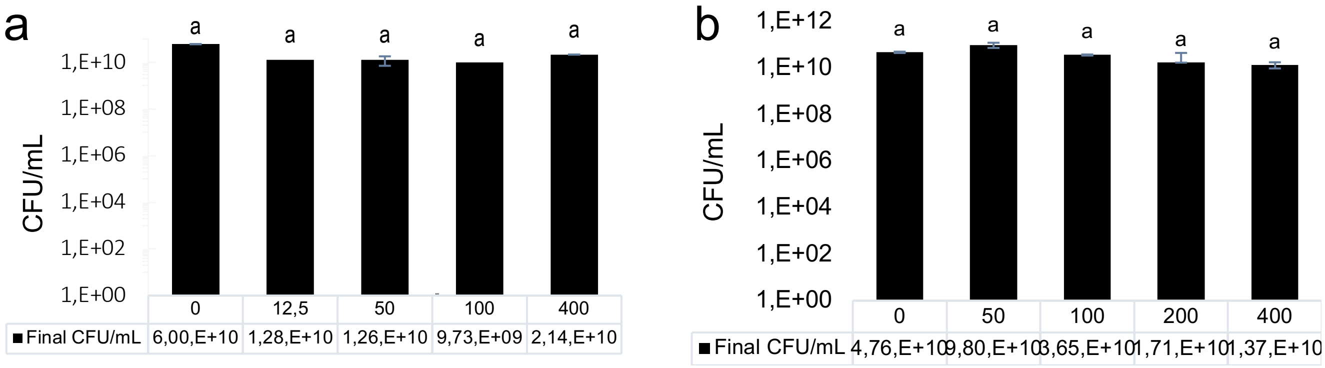

Error bars represent standard errors of the mean (n = 3). Bars sharing letters do not differ statistically in their values detected by Kruskal-Wallis rank test and Post-hoc Duncan-test for multiple comparisons (a) *P- value* = 4.11 E-1 b) *P-value* = 7.25 E-2)

Figure S6. Growth curves of *R. solanacearum* EAP09 and *B. cereus* EA-CB1047 in a co-culture in TTC presence and absence. Intervals represent standard errors of the mean (n = 3). Co-culture: indicates cultures of *R. solanacearum* and *B. cereus* simultaneously in the same flask; monoculture: indicates culture of *R. solanacearum* alone. (+TTC and -TTC): indicates in presence and absence of TTC respectively.

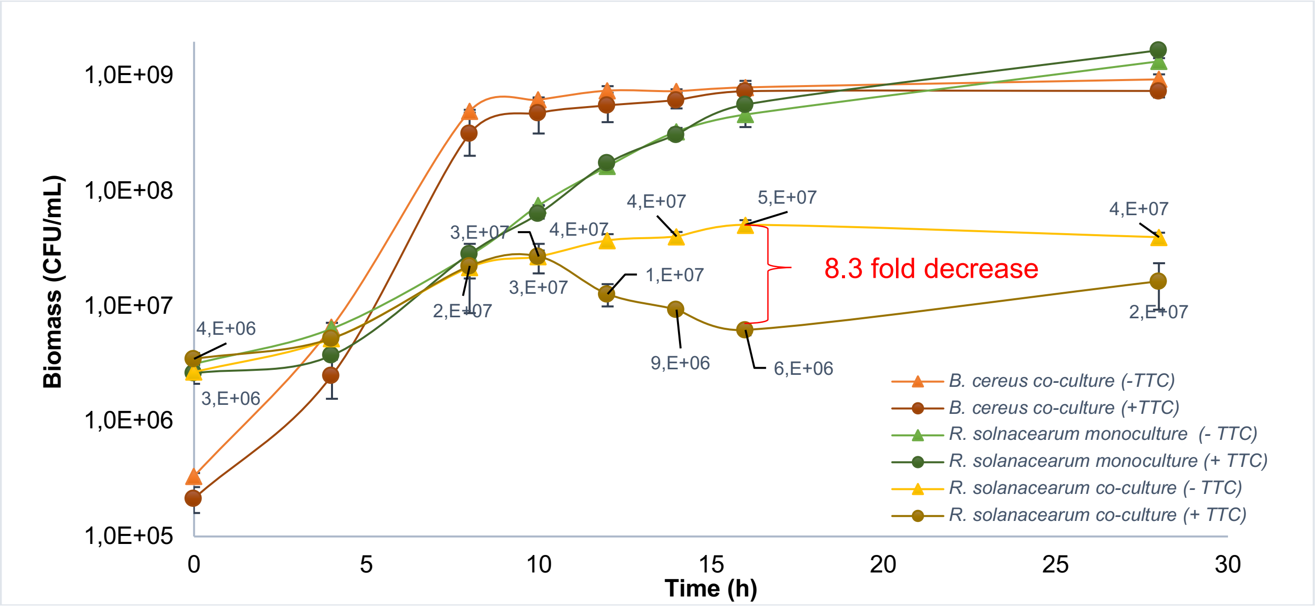

Figure S7. Siderophore production of *B. subtilis* NCIB-3610 assessed through CAS assay in presence and absence of TTC.

Methodology: Siderophore production by *Bacillus* sp. strains in the presence and absence of TTC was determined by the colorimetric assay established by Schwyn and Neilands (10). Briefly, pre-solutions of 10 mM hexadecyltrimethyl ammonium bromide (HDTMA, Sigma-Aldrich, MO, USA), 10 mM iron III in 1 N HCl and 0.12% Chrome Azurol S (CAS, Sigma-Aldrich, MO, USA) were prepared. Afterwards, blue agar was prepared by mixing in a 1:5 ratio, iron III acid solution with the aqueous CAS solution, before addition of a diluted (5 mM) HDTMA (Sigma-Aldrich, MO, USA) in a 1:1.5 ratio. This final solution (100 mL) was added to 900 mL of agar (15 g agarose, 32.24 g PIPES, 12 g NaOH). The trial consisted of placing agar discs of *Bacillus* sp. strains to test for siderophores production on top of BGA and BGTA plates, incubating for 24 h at 37ºC, afterwards overlaying with 15 mL of previously prepared blue agar. These plates were dried for 2 h and incubated for 24 h at 30ºC, until orange zones, which indicate ferric sequestering activity, were visible around bacterial colonies. Three replicates were used for each treatment.

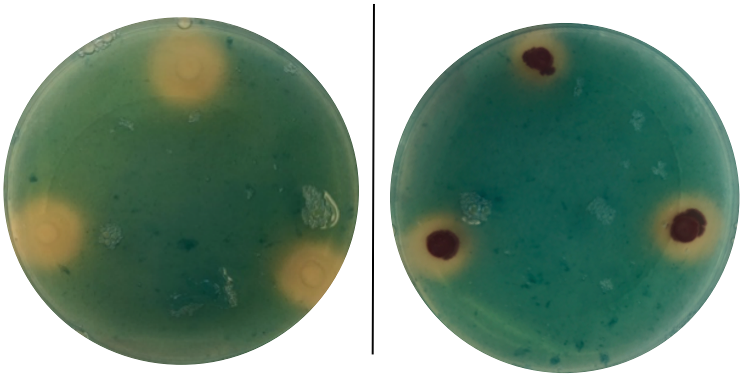

Figure S8. Effect of antioxidant compound on induced antagonistic capacity of *B. cereus* EA-CB1047 against *R. solanacearum* EAP-009.

Methodology: The effect of antioxidant compounds on the inducible activity was tested using the agar plug method. Final concentrations of 100 mg/L for ascorbic acid (vitamin C, Sigma-Aldrich, OH, USA), 10 mg/L for α-tocopherol (vitamin E, Sigma-Aldrich, OH, USA), 10 mg/L for uric acid (Sigma-Aldrich, OH, USA), and 18 mg/L for L-glutathione (Alfa-Aesar, Heysham, UK) were applied in BGA and BGTA plates. Three replicates were used for each treatment.

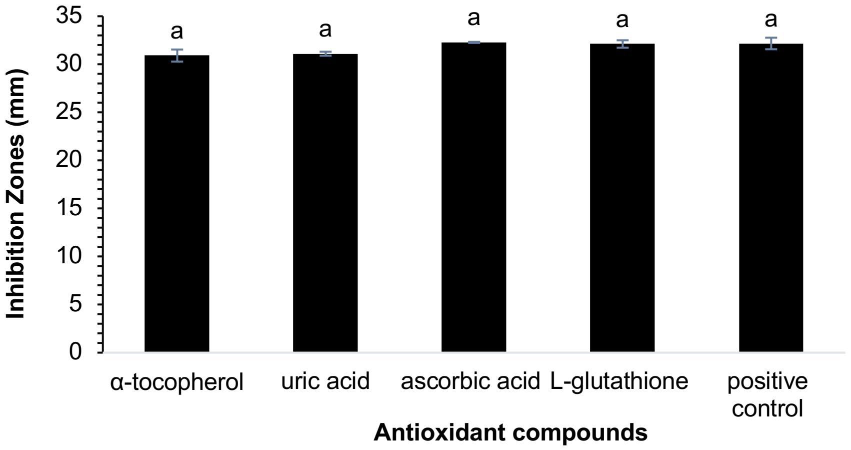

Intervals represent standard errors of the mean (n = 3) and no statistically significant differences were found among treatments (*P-value* = 0.1388) by one-way ANOVA. Final concentrations in culture medium for antioxidant compounds are: 10 ppm for α-tocopherol and uric acid, 100 ppm for ascorbic acid and 18 ppm for L-glutathione. Positive control consisted on a BGTA plate with 0 ppm of antioxidants.

References

1. The UniProt Consortium. UniProt: the universal protein knowledgebase. Nucleic Acids Res. 2017;45(D1):D158-D69.
2. Shannon P, Markiel A, Ozier O, Baliga NS, Wang JT, Ramage D, et al. Cytoscape: a software environment for integrated models of biomolecular interaction networks. Genome Res. 2003;13(11):2498-504.
3. Al-Ajlani MM, Sheikh MA, Ahmad Z, Hasnain S. Production of surfactin from Bacillus subtilis MZ-7 grown on pharmamedia commercial medium. Microb Cell Fact. 2007;6:17.
4. Chen H, Wang L, Su CX, Gong GH, Wang P, Yu ZL. Isolation and characterization of lipopeptide antibiotics produced by Bacillus subtilis. Lett Appl Microbiol. 2008;47(3):180-6.
5. Villegas-Escobar V, Ceballos I, Mira JJ, Argel LE, Orduz Peralta S, Romero-Tabarez M. Fengycin C produced by Bacillus subtilis EA-CB0015. J Nat Prod. 2013;76(4):503-9.
6. Lee JY, Passalacqua KD, Hanna PC, Sherman DH. Regulation of petrobactin and bacillibactin biosynthesis in Bacillus anthracis under iron and oxygen variation. PLoS One. 2011;6(6):e20777.
7. Miethke M, Klotz O, Linne U, May JJ, Beckering CL, Marahiel MA. Ferri-bacillibactin uptake and hydrolysis in Bacillus subtilis. Mol Microbiol. 2006;61(6):1413-27.
8. Özcengiz G, Öğülür İ. Biochemistry, genetics and regulation of bacilysin biosynthesis and its significance more than an antibiotic. N Biotechnol. 2015;32(6):612-9.
9. Wang M, Carver JJ, Phelan VV, Sanchez LM, Garg N, Peng Y, et al. Sharing and community curation of mass spectrometry data with Global Natural Products Social Molecular Networking. Nat Biotechnol. 2016;34(8):828-37.
10. Schwyn B, Neilands JB. Universal chemical assay for the detection and determination of siderophores. Anal Biochem. 1987;160(1):47-56.
